# A study of the heterochronic sense/antisense RNA representation in florets of sexual and apomictic *Paspalum notatum*

**DOI:** 10.1186/s12864-021-07450-3

**Published:** 2021-03-16

**Authors:** Maricel Podio, Carolina Colono, Lorena Siena, Juan Pablo A. Ortiz, Silvina Claudia Pessino

**Affiliations:** Instituto de Investigaciones en Ciencias Agrarias de Rosario (IICAR-CONICET-UNR), Facultad de Ciencias Agrarias, Universidad Nacional de Rosario, Campo Experimental Villarino, (S2125ZAA) Zavalla, Santa Fe, Argentina

**Keywords:** Apomixis, Apospory, Molecular breeding, Plant reproduction

## Abstract

**Background:**

Apomixis, an asexual mode of plant reproduction, is a genetically heritable trait evolutionarily related to sexuality, which enables the fixation of heterozygous genetic combinations through the development of maternal seeds. Recently, reference floral transcriptomes were generated from sexual and apomictic biotypes of *Paspalum notatum*, one of the most well-known plant models for the study of apomixis. However, the transcriptome dynamics, the occurrence of apomixis vs. sexual expression heterochronicity across consecutive developmental steps and the orientation of transcription (sense/antisense) remain unexplored.

**Results:**

We produced 24 Illumina TruSeq®/ Hiseq 1500 sense/antisense floral transcriptome libraries covering four developmental stages (premeiosis, meiosis, postmeiosis, and anthesis) in biological triplicates, from an obligate apomictic and a full sexual genotype. De novo assemblies with Trinity yielded 103,699 and 100,114 transcripts for the apomictic and sexual samples respectively. A global comparative analysis involving reads from all developmental stages revealed 19,352 differentially expressed sense transcripts, of which 13,205 (68%) and 6147 (32%) were up- and down-regulated in apomictic samples with respect to the sexual ones. Interestingly, 100 differentially expressed antisense transcripts were detected, 55 (55%) of them up- and 45 (45%) down-regulated in apomictic libraries. A stage-by-stage comparative analysis showed a higher number of differentially expressed candidates due to heterochronicity discrimination: the highest number of differential sense transcripts was detected at premeiosis (23,651), followed by meiosis (22,830), postmeiosis (19,100), and anthesis (17,962), while the highest number of differential antisense transcripts were detected at anthesis (495), followed by postmeiosis (164), meiosis (120) and premeiosis (115). Members of the AP2, ARF, MYB and WRKY transcription factor families, as well as the auxin, jasmonate and cytokinin plant hormone families appeared broadly deregulated. Moreover, the chronological expression profile of several well-characterized apomixis controllers was examined in detail.

**Conclusions:**

This work provides a quantitative sense/antisense gene expression catalogue covering several subsequent reproductive developmental stages from premeiosis to anthesis for apomictic and sexual *P. notatum*, with potential to reveal heterochronic expression between reproductive types and discover sense/antisense mediated regulation. We detected a contrasting transcriptional and hormonal control in apomixis and sexuality as well as specific sense/antisense modulation occurring at the onset of parthenogenesis.

**Supplementary Information:**

The online version contains supplementary material available at 10.1186/s12864-021-07450-3.

## Background

Apomixis (i. e., agamospermy) is an asexual mode of plant reproduction via seeds, which generates progenies consisting of exact genetic replicas of the mother plant [1]. This puzzling trait occurs in at least 326 angiosperm genera, with no clear tendency to any specific group [2]. The apomictic and sexual developmental pathways are strongly related, since both take place within the ovule and involve common developmental features [3]. Traditionally, the asexual route was considered to be a deviation from the sexual one, repeatedly emerging during evolution from genetic or epigenetic mutations derived from polyploidization and/or hybridization events [4–6]. More recently, apomixis and sexuality were hypothetically classified as ancient polyphenic traits [7]. According to the latter view, the switching from one phenism to the other would be environmentally triggered by epigenetic mechanisms, with full sexual genera/species having lost the capacity to carry on this transition due to deleterious (epi)mutations affecting the molecular switch that connects both pathways [7].

The potential of apomixis for fixing heterosis has long been recognized [8, 9]. Therefore, the use of this trait in combination with sexuality can dramatically accelerate the development of new hybrid varieties and reduce the costs associated with seed-production [10]. Among other advantages, the transference of apomixis to species of agricultural interest will allow the perpetuation of genetic resources including wide-cross hybrids, the rapid generation of adapted crops, the avoidance of monocultures and the development of maternal seeds from vegetatively propagated cultivars, like potatoes or strawberries [10–12]. The apomixis breeding technology impact in global agriculture could be comparable to that produced by the green revolution, initiated in USA, Mexico, India and further spread to other countries in the middle ‘70s [13].

While plant sexuality takes off with a specialized cell division process (meiosis) preceding the formation of haploid megaspores, apomictic mechanisms share the common characteristic of lacking any reductive division before female sporogenesis (apomeiosis) [1]. Moreover, while sexuality start the sporophytic life cycle by restoring the species-specific ploidy level through fertilization of the genetically reduced egg cell with an equally reduced male gamete, apomixis does it by inducing parthenogenesis, i. e., the spontaneous formation of an embryo from a reproductively-committed cell [1]. Finally, for the formation of the seed endosperm, the sexual route requires the fertilization of two reduced female polar nuclei with a reduced male gamete under a strict 2:1 maternal:paternal genomic contribution. Instead, apomixis may alternatively proceed with the spontaneous proliferation of maternal polar nuclei (autonomy) or the fertilization of one/two female unreduced polar nuclei with a male reduced gamete (pseudogamy), a path that often deviates from the expected genome contribution ratio [1]. While in some species the three components of apomixis (apomeiosis, parthenogenesis and endosperm development) seem to be controlled by a non-recombinant superlocus, in others these factors can be readily uncoupled [2, 3, 14–16]. However, in nearly all cases there seem to be a consistent association between the expressivity of trait and the ploidy level increments [2, 3, 14–16].

In the past decade, transcriptome surveys have exposed a large number of genes differentially regulated in sexual and apomictic developmental pathways. Analyses on *Boechera spp.* [17, 18]; *Boehmeria tricuspis* [19]; *Brachiaria spp.* [20, 21]; *Citrus spp*. [22]; *Eragrostis curvula* [23]; *Hieracium praealtum* [24]; *Hypericum perforatum* [25]; *Poa pratensis* [26]; *Panicum maximum* [27]; *Paspalum spp.* [28–31]; and *Pennisetum spp.* [32–35] used different strategies to reveal the molecular cohorts modulating the trait. Although divergent evolution of apomictic species validates a partially contrasting nature for the detected candidate transcripts, some pathways do seem consistently altered, especially those related to cell cycle and cell division control, ribosome metabolism, RNA processing, signal transduction, hormone signaling and epigenetic mechanisms [36]. Moreover, long non-coding and antisense RNAs [37–41], as well as, small RNAs that target specific transcription factors [22, 42–45] are being increasingly recognized as common members of the apomixis cascade.

*Paspalum notatum* Flüggé is a warm-season perennial grass widely distributed in the Western Hemisphere [46], where it occurs as a primary constituent of natural grasslands, particularly in southern Brazil, Paraguay, Uruguay, and north-east Argentina [47]. The species form a multiploid complex in which the diploid cytotype (2n = 2x = 20) is self-sterile and sexual, while the tetraploid one (the common race) (2n = 4x = 40) is pseudogamous, aposporous apomictic and self-fertile [48]. Other infrequent polyploid cytotypes (3x and 5x) are also apomicts [49]. Moreover, numerous sexual tetraploid individuals were artificially synthetized from diploids by colchicine treatment, or obtained from experimental crosses involving facultative apomicts [50]. Interestingly, although sexual seeds form the endosperm under a strict 2 maternal: 1 paternal genomic contribution, apomictic ones are more permissive and can develop under different maternal:paternal genomic ratios (e.g., 4:1; 8:1) [49]. *P. notatum* has become a model system for apomixis research and breeding, mainly due to the existence of freely-crossable races of the same ploidy and different reproductive mode, a thoroughly-characterized living germplasm collection and advanced breeding programs exploiting apomixis for cultivar generation [48, 50–53].

Several transcriptome surveys were conducted to identify *P. notatum* apomixis-associated genes. Firstly, transcripts expressed in sexual and apomictic florets were compared by using differential display, allowing the identification of a pioneer apomixis candidate gene homologous to the maize kinesin-like motor protein KIN-14P [28]. Then, Laspina et al. [29] identified 65 transcripts differentially expressed in spikelets of apomictic and sexual genotypes at premeiosis/meiosis, several of which mapped in silico to a rice chromosome 2 region that had previously been associated with apospory by comparative mapping [54, 55]. Moreover, endosperm RNA representation comparisons 3–24 h after pollination revealed more than 100 differentially expressed transcripts (DETs) in seeds that differed from the expected 2 m:1p genome contribution ratio, formed when apomictic plants were used as female parents in crosses (the endosperm involved 4 m:1p, 8 m:1p or 8 m:3p contribution ratios, depending on the cross) [56]. Besides, transcripts related to endosperm development were identified in apomictic and sexual ovaries of *Paspalum notatum* 48 h after pollination, a stage prior to post-zygotic collapse [57]. These DETs were mainly associated with genes related to transcription, signal transduction, growth/division, protein destination and storage, as well as regulation of gene expression. Interestingly, several differential sequences identified at the onset of endosperm development showed high similarity with proteins expressed in response to changes in the levels of extracellular ATP [56, 57]. Besides, the Roche 454/FLX + long-read technology was used to produce apomictic and sexual reference floral transcriptomes on an equitable mix of RNA extracted from spikelets at different developmental states from premeiosis to anthesis [31]. Recently, De Oliveira et al. (2020) [58] reported a global gene expression analysis using Illumina Hi-Seq technology on RNA isolated from leaf and floral tissues of 2x sexual, 4x sexual and 4x apomictic genotypes. Interestingly, 89 DETs expressed in apomictic or sexual plants mapped at the chromosome regions of rice syntenic to the Paspalum apomixis controlling locus (ACL) [58].

All the above-cited contributions have partially disclosed the nature of numerous apomixis candidates and evidenced the effects of polyploidy on gene expression. However, our knowledge on the chronological modulation of transcript levels along the sexual and the apomictic reproductive routes remained limited, since previous studies were conducted on samples collected at a particular timeframe or on mixed pools representing a group of developmental stages. Under these experimental limitations, either part of the DETs go undetected or those experimenting expression increments at different developmental stages for contrasting reproductive modes are mistakenly classified as non-differential. Moreover, the plus/minus orientation of the expressed transcripts remains globally unexplored, even when some apomixis candidates (*ORC3*, *PsACR/H5*, *PsACR/H.13*) display antisense differential expression [39, 59]. The lack of comprehensive data prevents researchers from grasping the true dimensions of heterochronic expression and antisense regulation affecting apomixis development as well as from inspecting their biological consequences. The objective of this work was to gain a quantitative, statistically significant, massive characterization of sense/antisense transcripts expressed across four crucial reproductive steps in sexual and apomictic *P. notatum* and, after reciprocal comparisons, produce a detailed picture of the main molecular pathways operative during apomixis.

## Results

### Sequencing and de novo assembly

**Fig. 1:**
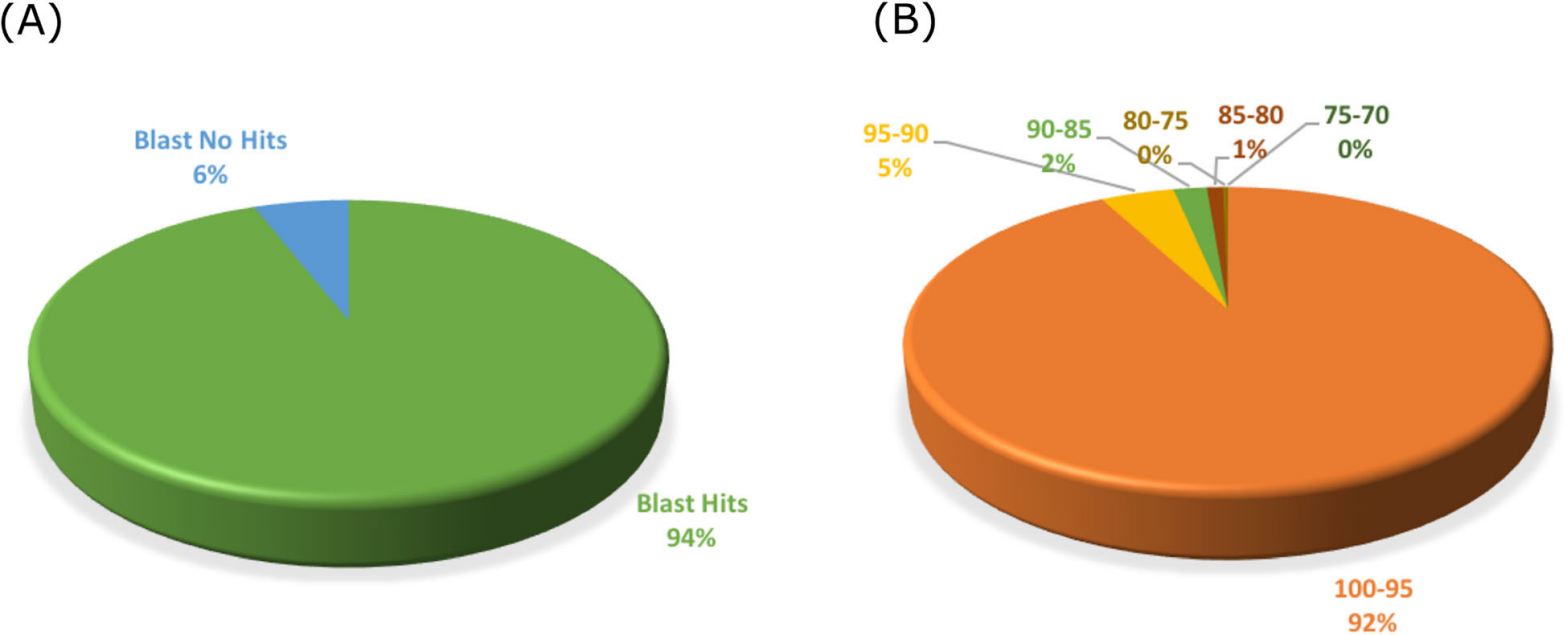
Statistics of the BLASTN comparison between the *P. notatum* Illumina HiSeq GTA and the Roche 454/FLX + global assembly. **a** Percentage of GTA transcripts with a significant hit (green) or no hit (blue) in the Roche 454/FLX + reference [31]. **b** Distribution of the identity percentages in the BLASTN analysis

To get a compressive characterization of sense/antisense transcripts expressed during the *P. notatum* reproductive development, Illumina TruSeq floral cDNA libraries representing two reproductive modes (apomixis and sexuality) and four developmental stages (premeiosis, meiosis, postmeiosis, anthesis), each one including three biological replicas, were sequenced with Illumina HiSeq 1500 technology. The procedure involved 24 libraries and generated a total of 60.94 Gb, of which 97% had a Phred value > 30. After demultiplexing, 292,647,558 pass filter (PF) reads were selected, which, after cleaning and trimming, yielded 234,957,559 high-quality reads (Q score > 30) (available under the NCBI SRA accession PRJNA511813). De novo assemblies were carried out with the Trinity software [60, 61], considering two groups of samples (apomictic and sexual), each of them containing 12 libraries. In a first trial, the available Roche 454/FLX + *P. notatum* reference floral transcriptome [31] was used as a guide reference. Then, a second de novo assembly, without any reference, was constructed to detect novel transcripts. Table 1 shows the output basic statistics derived from both procedures. As expected, the de novo assembly without a guide reference generated a higher number of contigs for both samples, probably reflecting the inclusion of low expressed transcripts and/or allelic variants absent in the Roche 454/FLX + transcriptome. Subsequently, the four assemblies were concatenated in one file and filtered to obtain 199,074 non-redundant transcripts corresponding to a global transcriptome assembly (GTA), representing both reproductive modes (apomixis and sexuality), and the four developmental steps (premeiosis, meiosis, postmeiosis, anthesis) (Table 1). This transcriptome shotgun assembly was deposited at DDBJ/EMBL/GenBank under the accession GIUR00000000. With an average length of 1182.31 bp and an N50 of 1508 bp, the assembled sequences resulted in good quality for annotation (Table 1). Mapping of raw reads onto the GTA with Bowtie2 and TopHat showed 99.09 and 98.3% of match with the reference, respectively. These results indicated the GTA covered almost the complete set of sequence reads. To validate the assembly identity, a BLASTN assay was performed onto the Roche 454/FLX + reference transcriptome [31]. Ninety-four % of the transcripts showed homology with the reference and 92% of them exhibited more than 95% identity (Fig. 1). A survey of the assembled contigs with TransDecoder identified 108,011 (54.24%) entities with protein-coding capacity, while BUSCO estimated a gene coverage of 93.1% (35.6% single copy and 57.5% duplicated genes). This value was higher than the coverage achieved with the Roche 454/FLX + reference transcriptome [31], which rounds about 82.2% (Additional file 1).

**Table 1:** Statistics of *Paspalum notatum* assemblies

Statistics	Assemblies				
	de novo with Roche 454/FLX + as guiding reference		de novo without reference		GTA^**a**^
	Sex	Apo	Sex	Apo	All
**Number of contigs**	80,177	79,040	100,114	103,699	199,074
**%GC**	50.32	49.82	48.60	48.17	48.73
**Median contig length**	781	791	980	903	897
**Average contig**	1012.51	1023.35	1285.99	1186.29	1182.31
**N50**	1199	1218	1696	1528	1508
**Number of contigs**	81,179,923	80,885,743	128,745,833	123,016,839	235,450,926

^a^Global transcriptome assembly

### Global transcriptome assembly (GTA) annotation

**Fig. 2:**
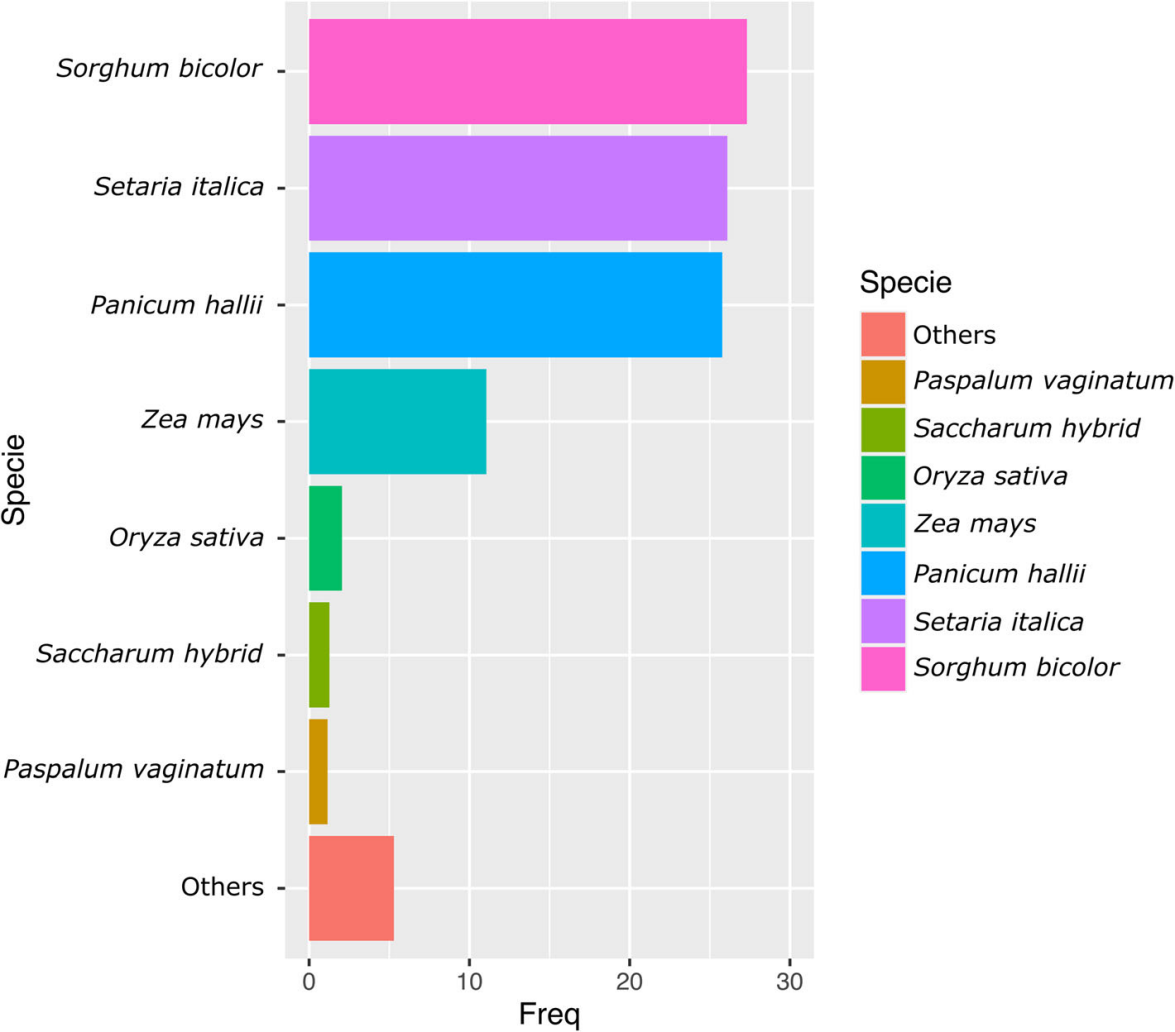
Species of origin distribution of top hits revealed by the GTA vs. NCBI (NT database) BLASTN surveys. The majority of the sequences matched at best with *Sorghum bicolor*, *Setaria italica* and *Panicum hallii* sequences. As expected, most hits correspond to gramineae species

A total of 101,079 transcripts produced robust top BLASTN hits against the NCBI NT database (E-value: 1e^− 15^; % query coverage > 30), and more than 98% of them matched to monocot sequences, mainly corresponding to *S. bicolor*, *S. italica*, *P. hallii* and *Z. mays* (Fig. 2). Besides, more than 96,350 transcripts showed significant hits against the UniProt database (taxon identifier: 58,024, BLASTP, E-value: 1 x e^− 5^). In total, 66,617 transcripts were grouped into 755 Cellular Component-, 88,776 into 558 Biological Process-, and 15,620 into 147 Molecular Function-GO terms. Additional file 2 (A-C) shows the 30 most representative GO terms for each category. Besides, 43,275 transcripts were annotated into 168 Kyoto Encyclopedia of Genes and Genomes (KEGG) pathways, with carbon metabolism, biosynthesis of amino acids, spliceosome, endocytosis, mRNA surveillance and glycolysis/gluconeogenesis as the most represented routes (Additional file 2, D).

### Apomixis vs. sexuality differential expression analyses

Based on the GTA, we analyzed the expression level of the complete set of sense and antisense transcripts by using the DESeq2 package. Comparisons included: *i*) a global differential expression analysis (GDEA), in which pairwise comparisons concerned the reproductive modes (apomictic vs. sexual) considering all developmental stages together (i.e., involving reads generated from all developmental stages), and *ii*) a stage-specific differential expression analysis (SSDEA) between reproductive modes at each developmental phase (i.e., involving reads generated from each particular developmental stage).

From the 199,074 total contigs, 188,823 passed the filters established for the GDEA (see Materials and Methods) and revealed 19,352 DETs with highly significant differential expression (False Discovery Rate: FDR < 0.001, Log_2_FC > |3|) between apomictic and sexual samples (Additional file 3). Most of the them (13,205; 68%) resulted upregulated in apomictic libraries (positive Log_2_FCs), while only 6147 (32%) were upregulated in sexual ones (negative Log_2_FCs) (Additional file 4). A heat map based on transcripts per million (TPM) values shows the general picture of sense transcripts expression patterns in both apomictic and sexual libraries (Additional file 5, A). Similar analyses involving antisense reads showed they targeted only 11,417 transcripts (6.04%) and, in most cases, covered only part of the sequences. The GDEA performed with these sequences (based on the number of reads mapped onto the GTA) showed only 100 differentially expressed antisense transcripts (DEATs) (FDR < 0.05, Log_2_FC > |3|). Fifty-five of them (55%) were upregulated in the apomictic libraries and 45 (45%) in the sexual one (Additional files 6 and 7). A heat map showing the number of counts mapped onto the differentially represented transcripts revealed the general antisense expression patterns based on TPM values for both apomictic and sexual libraries (Additional file 5, B). A comparison among DETs and DEATs revealed 18 transcripts common to both differential expression analyses.

**Fig. 3:**
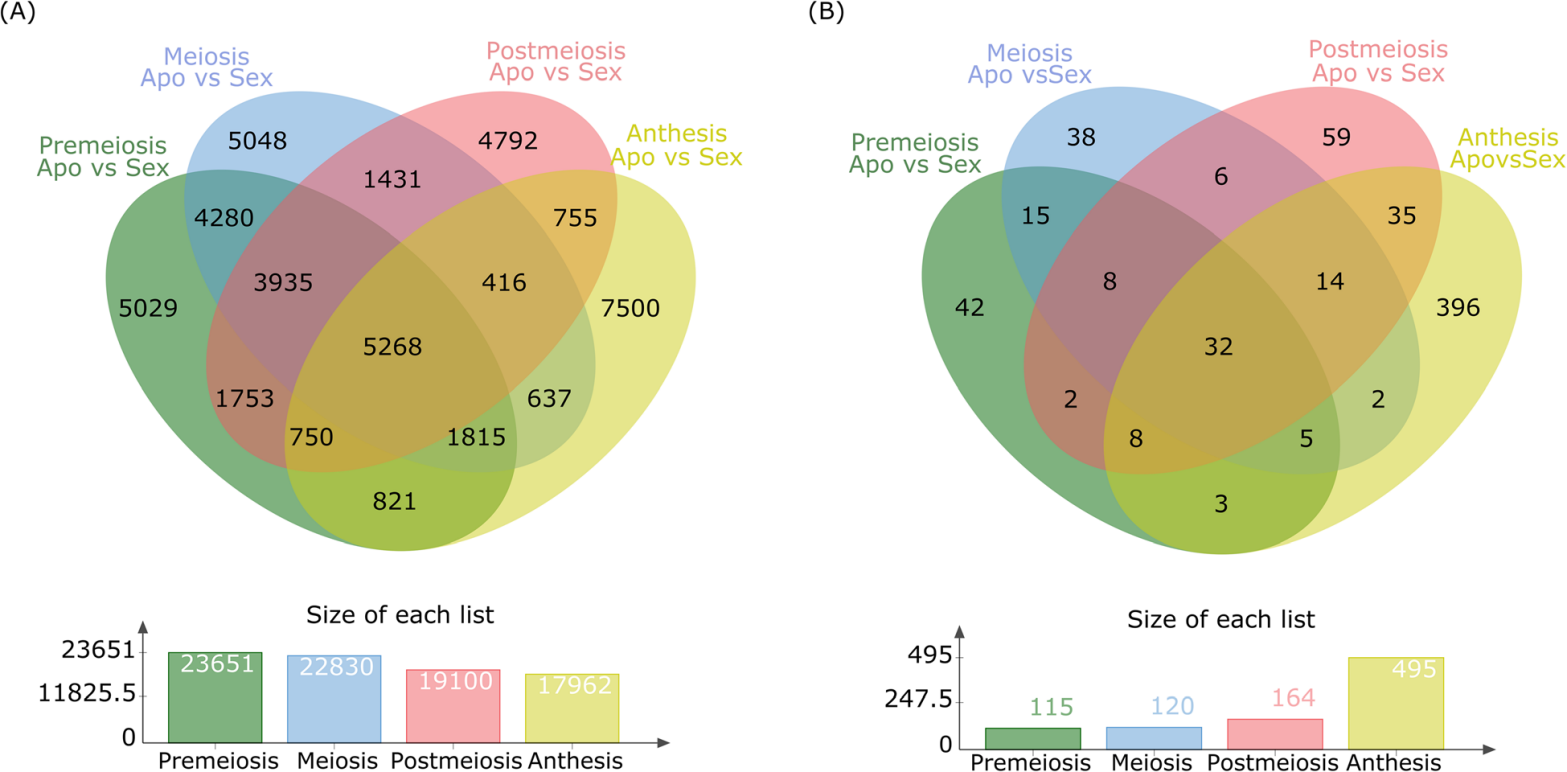
Venn diagrams displaying differential expression overlapping across developmental stages. **a** DETs occurring at different developmental stages. A total of 5268 DETs are differentially represented at all developmental stages. The developmental stage with the largest amount of stage-specific DETs is anthesis (7500). The developmental step with a largest amount of total DETs is premeiosis (23,651). **b** DEATs occurring at different developmental stages. A total of 32 DEATs are differentially represented at all developmental stages. The developmental step with a largest amount of stage-specific DEATs is anthesis (396). The developmental step with a largest amount of total DEATs is anthesis (495)

In the stage-specific differential expression analysis (SSDEA), the filtered raw reads corresponding to each developmental stage (premeiosis, meiosis, postmeiosis, anthesis) were mapped onto the GTA and compared between apomictic and sexual libraries. The highest number of DETs occurred at premeiosis, where, out of 124,569 expressed transcripts, 23,651 (FDR < 0.001, Log_2_FC > |3|) showed differential representation (Additional file 8, S1). At meiosis, out of 131,993 total transcripts, 22,830 DETs were identified (Additional file 8, S2), while at postmeiosis and anthesis, 19,100 (from 139,643) and 17,962 (from 139,460) transcripts were found differentially expressed, respectively (Additional file 8, S3 and S4). Considering the percentage of DETs overexpressed in apomictic plants, the largest number was detected at meiosis (72%) followed by premeiosis (69%), postmeiosis (68%), and anthesis (56%), showing the same tendency observed in the GDEA analysis, in which upregulated transcripts were more abundant in apomicts. A Venn diagram showing the number of transcripts with a significantly different level of expression between the apomictic and the sexual libraries at each developmental stage is presented in Fig. 3a. This analysis showed 5268 DETs common to all developmental stages (Fig. 3a). Nevertheless, a considerable proportion of the DETs appeared to be stage-specific. For instance, 5029 (21.30%) DETs were differentially expressed only at premeiosis. Likewise, at meiosis, postmeiosis and anthesis, other 5048 (22.11%), 4792 (25.01%) and 7500 (41.75%) stage-specific DETs were detected, respectively. Interestingly, the highest proportion of stage-specific DETs was detected at anthesis, when parthenogenesis start in most apomictic ovaries. A similar analysis carried out for the antisense transcripts showed 115, 120, and 164 DEATs (FDR < 0.05; Log_2_FC > |3|) at premeiosis, meiosis and postmeiosis, respectively (Additional file 9, S1-S3). The largest number of DEATs corresponded to anthesis, since 495 transcripts carrying antisense sequences were detected (Additional file 9, S4). Once again, the number of upregulated DEATs was higher than the downregulated ones at premeiosis (59% vs. 41%), meiosis (63% vs. 37%) and postmeiosis (54% vs. 41%). The opposite behavior was observed at anthesis, with 203 (41%) overrepresented and 292 (59%) downrepresented transcripts in apomictic libraries. A Venn diagram showing stage-specific DEATs is provided in Fig. 3b. Thirty-two (32) DEATs common to the four stages of development were observed (Fig. 3b). Furthermore, 42, 38, 59 and 396 stage-specific DEATs (i. e., occurring only at one particular stage) were detected at premeiosis, meiosis, postmeiosis, and anthesis, respectively. Here again, a large proportion of stage-specific transcripts, in this case antisense ones, occurs at anthesis, concurrent with the onset of parthenogenesis (Fig. 3b).

### Gene ontology and KEGG pathway classification

**Fig. 4:**
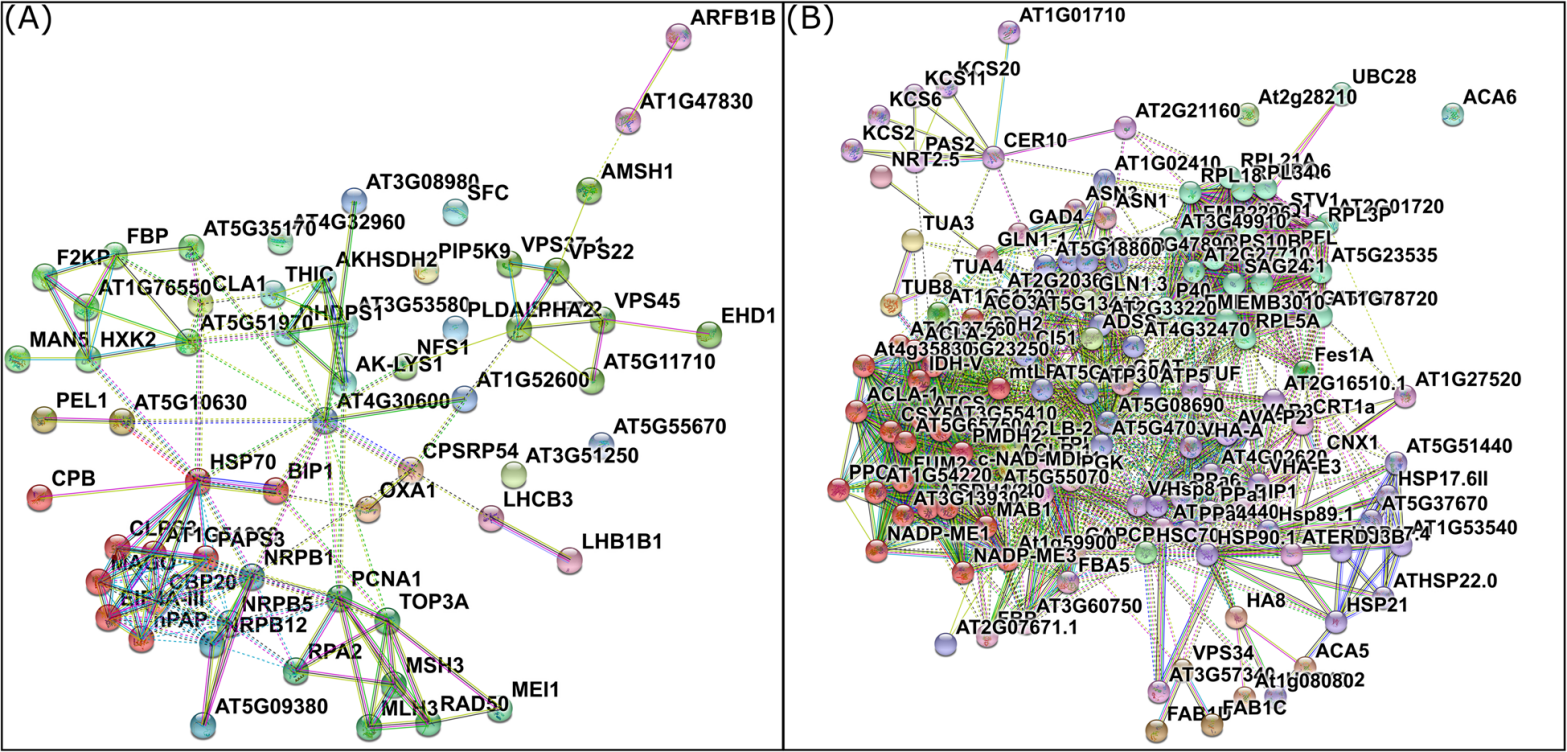
Molecular interaction networks for premeiosis and anthesis stages. The interaction predictions were established with the STRING database, based on DETs involved in the stage-specific KEGG pathways for: **a** premeiosis (onset of apospory initials) and **b** anthesis (parthenogenesis)

Next, we established a Gene Ontology (GO) classification for all sense transcripts expressed at each developmental stage (Additional file 8). Based on this catalogue, we carried out a KEGG pathways prediction to identify specifically-modulated molecular routes (i.e., upregulated or downregulated in apomictic plants) (Additional file 10). Homologous recombination, endocytosis, thiamine metabolism, monobactam biosynthesis, lysine biosynthesis, protein export, RNA polymerase, photosynthesis-antenna proteins, mismatch repair, mRNA surveillance pathway, fructose and mannose metabolism, various types of N − glycan biosynthesis, terpenoid backbone biosynthesis and phenylalanine metabolism pathways are regulated (by upregulation or repression) only at premeiosis, a clear turning point in which the onset of apospory initials occurs (Additional file 10). In the rest of the developmental stages these routes are similarly expressed in flowers of apomictic and sexual plants. Besides, mitogen-activated protein kinase (MAPK) signaling pathways, glycosphingolipid biosynthesis and galactose, pyrimidine, sphingolipid and biotin metabolisms are modulated exclusively during meiosis. Moreover, valine, leucine and isoleucine degradation, ABC transporters, circadian rhythms, autophagy, plant-pathogen interaction, fatty acid metabolism, aliphatic and aromatic aminoacid biosynthesis and selenocompound metabolism are regulated exclusively at postmeiosis. Finally, protein processing in the endoplasmic reticulum, ribosome, citrate cycle, oxidative phosphorylation, phagosome, nitrogen metabolism, alanine, aspartate and glutamate metabolism, carbon fixation in photosynthetic organisms and fatty acid elongation are modulated only at anthesis, when parthenogenesis starts. Protein interaction predictions among members of these stage-specific routes analyzed by comparisons with the STRING database [62], revealed they integrate tight networks worth to get functionally explored (Fig. 4).

A similar GO analysis was conducted for DEATs (Additional files 9 and 10), but this time up- and down-regulated entities were not distinguished due to the low number of candidates involved. We were able to identify sequences involved in mRNA surveillance and ubiquitin-mediated proteolysis modulated only at premeiosis; others related to cyanoaminoacid metabolism modulated during postmeiosis; and pentose/glucuronate interconversions, vitamin B6 metabolism, flavonoid biosynthesis, ether lipid metabolism and fatty acid elongation occurring exclusively at anthesis. Interestingly, DEATs related to endocytosis and spliceosome resulted exclusively regulated at two developmental stages: meiosis and anthesis (Additional files 9 and 10).

Then, Gene Ontology (GO) Enrichment analyses were performed on a subset of 5268 DETs which were common to the four developmental stages analyzed (i.e., transcripts that were found differentially expressed at all stages of sexual and apomictic developments) (Additional file 11). The most represented GO terms regarding Cellular Component (CC) were ribonucleoprotein complex, nuclear lumen, vacuolar membrane, vacuolar part and chloroplast thylakoid. The most represented Biological Process (BP) terms were protein localization to organelle, the establishment of protein localization to organelle, nucleotide biosynthetic process, nucleoside phosphate biosynthetic process and pyruvate metabolic processes. The Molecular Function (MF) main classes were structural molecule activity, structural constituent of ribosome, adenyl nucleotide binding, adenyl ribonucleotide binding and ATPase activity. Moreover, the main KEGG-predicted pathways were ribosome, carbon metabolism and spliceosome (Additional file 11).

The same study was applied to common DEATs. In this case, out of 32 total transcripts, only a few could be assigned to GO terms corresponding to CC (nucleus), MF (zinc ion binding, stearoyl-CoA9-desaturase activity, ATP binding and amylase activity) and BP (mature ribosome assembly) (not shown). Although the limited number of DEATs did not allow a proper KEGG pathway evaluation, we could identify that fatty acid metabolism, MAPK signaling pathway, starch/sucrose metabolism and plant hormone signal transduction pathways were changed (not shown).

### Transcriptome dynamics

**Fig. 5:**
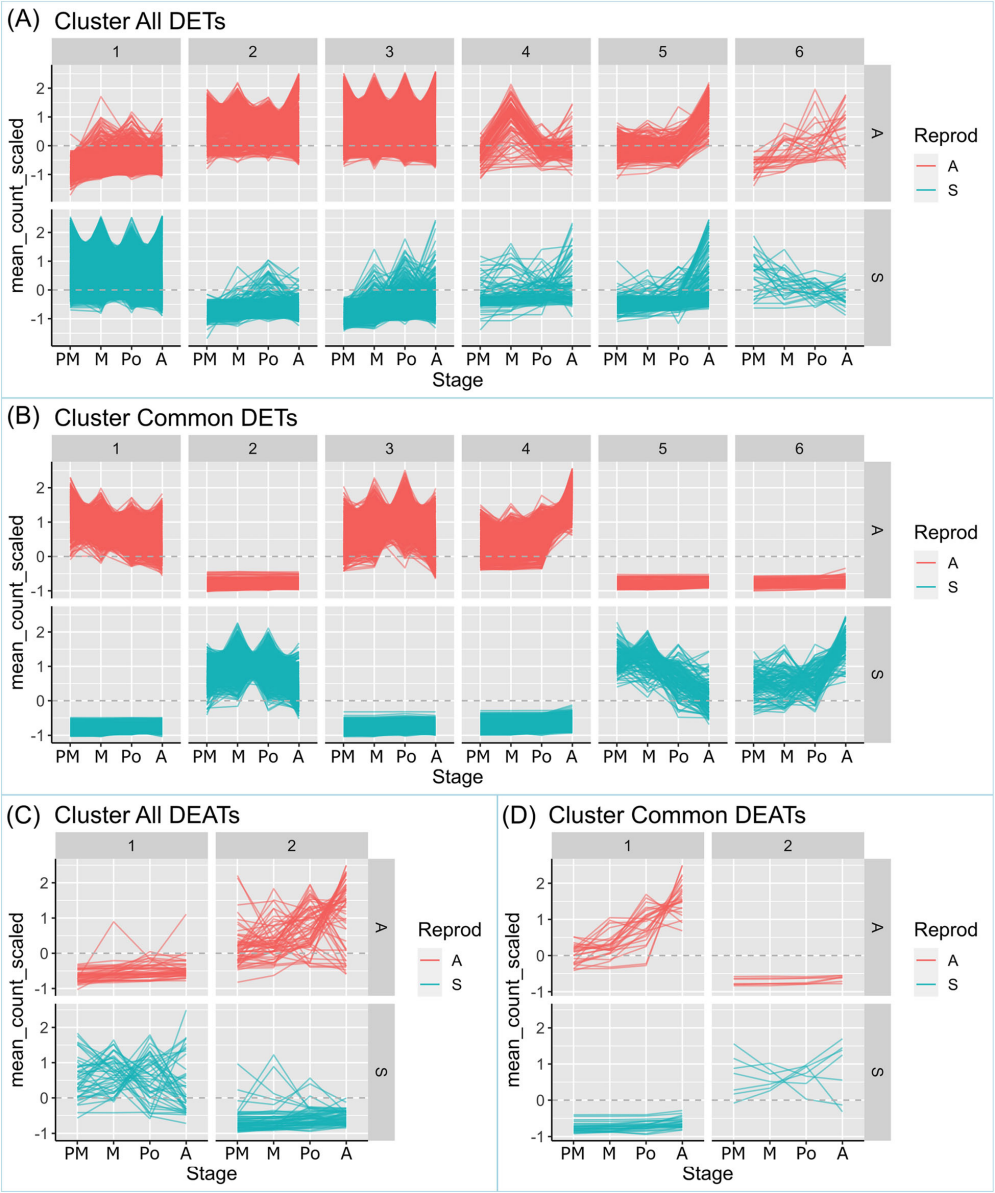
Transcript expression dynamics in clusters of selected genes. Clusters of transcripts showing similar expression, derived from: **a** the total (all) DETs raw counts matrix; **b** the common DETs raw counts matrix (common DETS are sense transcripts differentially expressed at all stages); **c** the total (all) DEATs raw counts matrix; **d** the common DEATs raw counts matrix (common DEATs are antisense transcripts differentially expressed at all stages). Vertical axis: mean counts (scaled) of the triplicate libraries. Horizontal axis: developmental stages: P: premeiosis; M: meiosis; Po: postmeiosis; A: anthesis

Matrixes representing the normalized raw read counts for each transcript in each one of the libraries are provided in Additional file 12 (S1 and S2 for sense and antisense transcripts, respectively). The advantage of counting with samples representing different developmental stages allowed us to perform a cluster analysis to identify groups of transcripts with similar expression patterns (Fig. 5). Normalized counts were used to execute a hierarchical clustering using a simple euclidean distance metric and a complete linkage method, to find some structure in our transcript expression trends and consequently partition our transcripts into different groups. To enable the analysis, subsets of transcripts were used, corresponding to: 1) all DETs and DEATs; 2) transcripts that were differentially expressed at all the developmental stages (common DETs and DEATs). 3) stage-specific DETs and DEATs (premeiosis, meiosis, postmeiosis and anthesis). The number of transcripts within each cluster is displayed in Table 2.

**Table 2:** Number of transcripts included in each cluster with similar expression pattern

Cluster	All_DE	Common	Premeiosis	Meiosis	Postmeiosis	Anthesis
**DETs (sense transcripts)**						
1	6068	1542	2225	1871	1593	1742
2	1621	612	1682	2730	2474	5239
3	11,314	2008	536	295	355	305
4	100	854	191	55	225	130
5	222	141	376	46	76	61
6	27	111	19	51	69	23
**Total**	19,352	5268	5029	5048	4792	7500
**DEATs (antisense transcripts)**						
1	42	25	24	21	28	269
2	58	7	18	17	31	127
**Total**	100	32	42	38	59	396

Clustering analysis split all DETs into six clusters displaying distinct expression patterns (Fig. 5a). Cluster 1 included transcripts up-regulated in the sexual samples at all stages (Fig. 5a). Clusters 2 and 3 showed transcripts up-regulated in apomictic samples at all stages, while clusters 4, 5 and 6 displayed variable divergent expression patterns (Fig. 5a). Particularly, Cluster 6 includes a group of transcripts showing expression heterochronicity, with entities overexpressed at postmeiosis/anthesis in apomictic plants but at premeiosis/meiosis in sexual ones (Fig. 5a). In the case of all DEATs, two major clusters were established (Fig. 5c). In Cluster 1, members show variable expression in sexual samples in the course of development, while in apomictic ones the expression seems lower or null (Fig. 5c). The opposite behaviour was observed in Cluster 2 (Fig. 5c).

Interestingly, the analysis of the common DETs (transcripts that were differentially expressed at all developmental stages) revealed a striking contrast between sample types (Fig. 5b). Clusters 1, 3 and 4, showed up-regulation in apomictic samples across the four developmental stages, while the same transcripts were consistently repressed in sexual ones (Fig. 5b). The opposite occurred in Clusters 2, 5 and 6 (Fig. 5b). In the case of common DEATs, Cluster 1 showed high expression increasing in the course of development in apomictic samples, but low modulated expression in sexual ones (Fig. 5d). The opposite occurred in Cluster 2 (Fig. 5d).

A similar study was conducted with the stage-specific DETs and DEATs. Clustering graphs are presented in Additional file 12 (S3 and S4, respectively). In all cases (premeiosis, meiosis, postmeiosis and anthesis), transcripts were organized into 6 different clusters with contrasting behaviour. Expression heterochronicity is clearly visible in clusters like DETs Postmeiosis 5 and DETs Anthesis 5.

### Differential expression of transcription factors

To investigate the nature, the level and the expression timing of transcription factors (TFs) detected in the apomictic and sexual samples, we contrasted our Illumina transcriptomes against the Plant Transcription Factor Database (http://planttfdb.cbi.pku.edu.cn). Based on the BLASTx top hits, we identified 63,076 (31.67%) transcripts highly similar to TFs, corresponding to 60 different families (29,847 of them with more than a 50% ID), several of which had previously been associated with reproductive development in *Arabidopsis* [63] (Additional file 13). The most abundant types corresponded to MYB, bHLH, NAC, WRKY, ERF, C2H2, FAR1, B3, C3H, bZIP, G2-like, M-type MADS, GRAS, LBD, TRIHELIX and ARF family proteins (Additional file 14, A). Comparative analyses showed that 6449 (10.22%) were DETs, of which 2078 were upregulated in sexual samples and 4371 in apomictic ones (Additional file 13). Afterwards, the number of upregulated TFs corresponding to each family was represented in a heat map (Additional file 14, B). Some families of TFs showed members upregulated at different developmental stages for apomictic or sexual plants (Additional file 13, Additional file 14, B). For instance, the MYB family shows a high number of members upregulated at premeiosis in apomictic libraries, while a few members are upregulated at anthesis in sexual libraries. Some families, like MYB, bHLH, ERF, WRKY, B3, ARF and AP2 showed a higher number of upregulated members in the apomictic samples across all developmental stages. Moreover, some families (like SAP and NZZ/SPL) presented up-regulated members only in the apomictic samples (not even one member was found upregulated in sexual plants). Moreover, others, like LFY, presented up-regulated members at all stages in the apomictic plant, but only at postmeiosis in the sexual genotype. An interesting case is that of LBD family proteins, which presented a higher number of upregulated members at anthesis in the sexual plant (Additional file 14, B).

### Differential expression of transcripts associated with plant hormones

A search for hormone-related transcripts expressed during the apomictic and sexual reproductive developments was performed by testing our transcriptomes against the *Arabidopsis* hormone-related protein database (http://hormones.psc.riken.jp/pathway_ja.html). A BLASTx analysis showed 3781 top hits associated with plant hormones and related compounds, including 714 related with auxin, 595 with jasmonate, 592 with cytokinin, 520 with abscisic acid, 517 with gibberellin, 487 with brassinosteroids, 245 with salicylic acid and 111 with ethylene (Additional file 15). Of them, 56 (related to auxin), 70 (related to jasmonate), 39 (related to cytokinin), 42 (related to abscisic acid), 46 (related to gibberellin), 36 (related to brassinosteroids), 21 (related to salicylic acid) and 5 (related to ethylene) transcripts resulted differentially expressed between the apomictic and sexual samples (DE Hormones) (Additional file 15, Fig. 6).

**Fig. 6:**
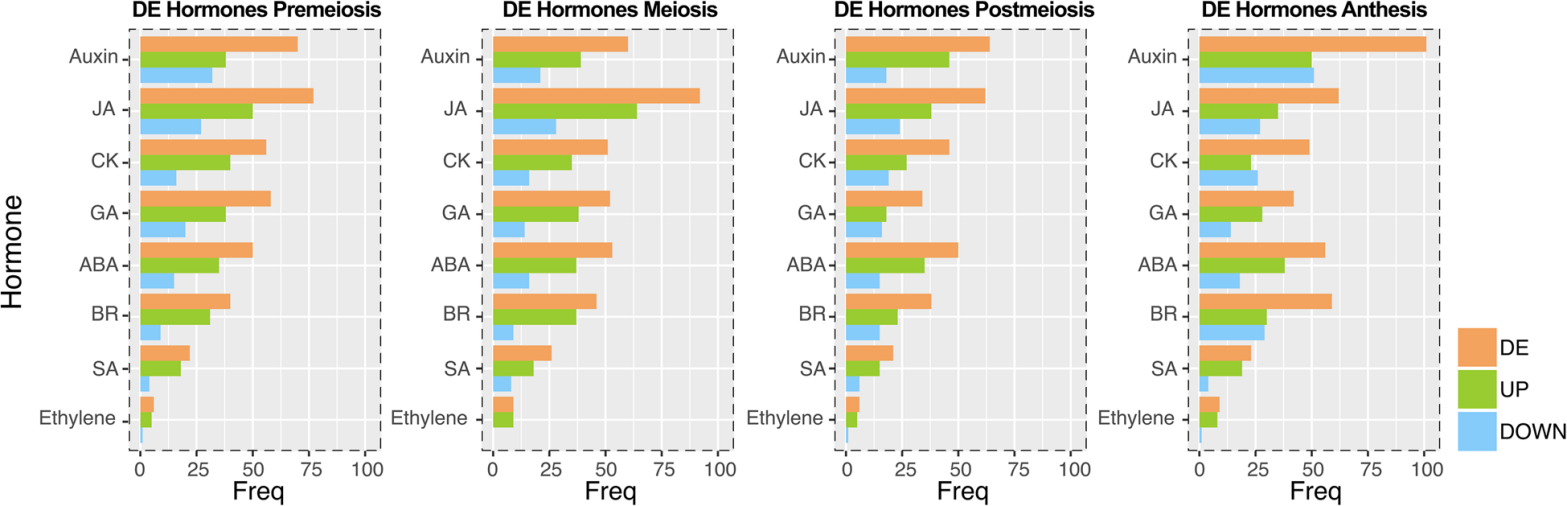
Plant hormones associated transcripts expressed from premeiosis to anthesis. Several hormonal routes are differentially regulated across the studied reproductive developmental steps (premeiosis, meiosis, postmeiosis and anthesis), with auxin and jasmonic acid pathways as the most represented. Both upregulated and downregulated members were detected in each case. DE Hormones stands for de-regulated hormone-related transcripts. DE: de-regulated. UP: up-regulated. DOWN: down-regulated. Ja: jasmonic acid. CK: cytokinin. GA: gibberelic acid. ABA: absicic acid. BR: brassinosteroids. SA:salycilic acid

### Apomixis candidates survey

**Fig. 7:**
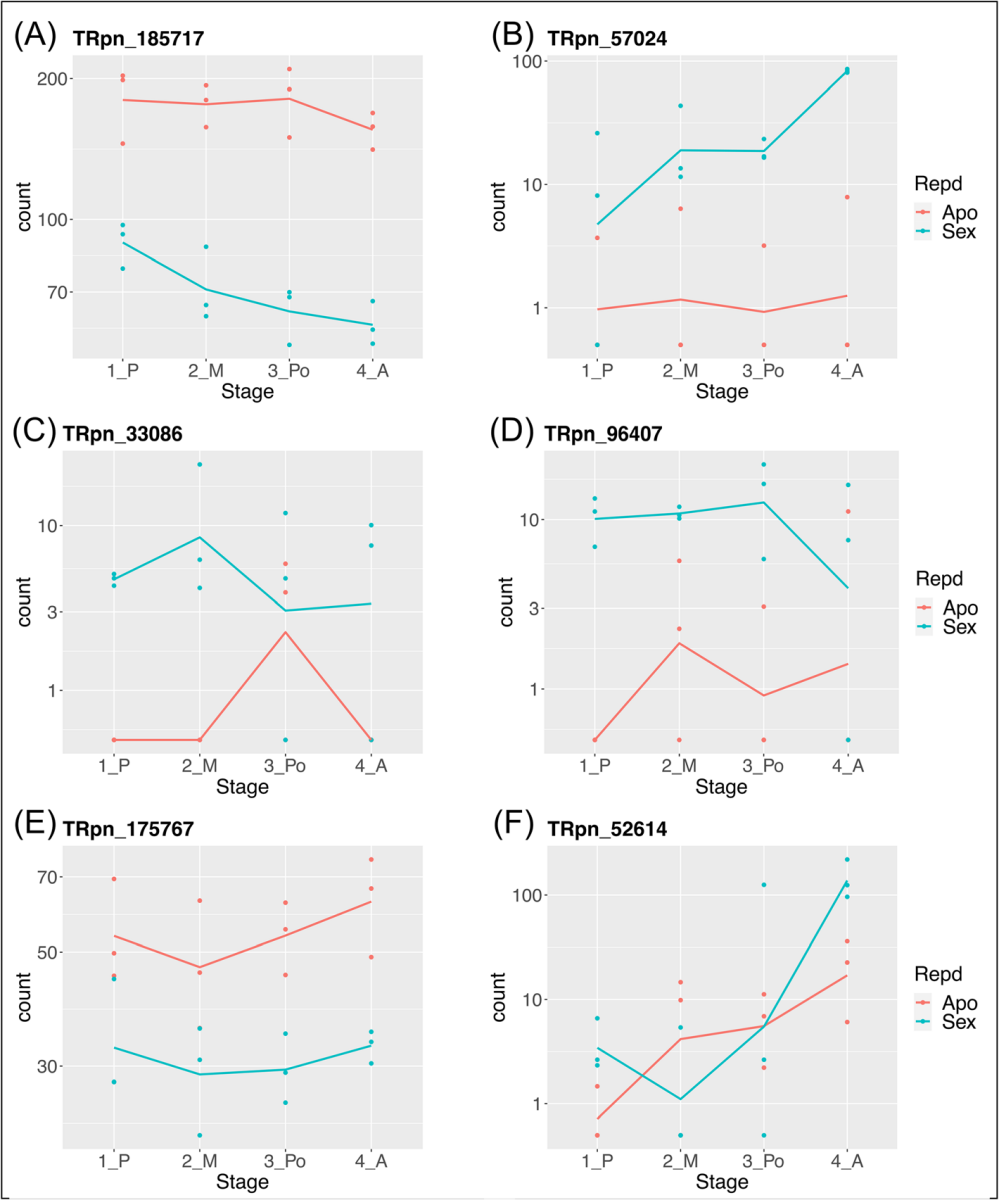
Expression patterns of apomixis-related transcripts across reproductive developmental stages from premeiosis to anthesis. **a** Argonaute 104 (TRpn_185717). **b** Gibberellin receptor GID1 (TRpn_57024), **c**, **d** ORC3b (TRpn_33086, TRpn_96407). **e**, **f** APOSTART2 (TRpn_175767, TRpn_52614). Repd: reproductive mode. Red lines: apomictic libraries. Blue lines: sexual libraries

In previous work, several genetically-linked, differentially-expressed or functionally-associated apomixis candidate genes were reported in *P. notatum* and other related species [36, 50, 58, 64]. Additional file 16 shows some of these candidate genes (with their respective identifiers) and 49 DETs displaying significant similarity them, as well as the stages at which differential expression is detected, the Log_2_FC and padj values for each developmental stage and the annotation of the sequence. Ocassionally, different DETs showed the same annotation but displayed contrasting expression profiles, pointing to the existence of transcripts variants of the same gene expressing at distinct reproductive stages. Ten (10) DETs showed different expression levels at all developmental stages, three were specific for premeiosis, five for meiosis, three for postmeiosis and nine for anthesis (Additional file 16). The rest revealed differential expression levels at more than one stage. The identities of DETs include, among others, *KIN-14P* [28], *ENHANCED DISEASE RESISTANCE 2 APOSTART1/2* [65], *FIE* [66], *LORELEI-like* (N20) [67], *SERK1/* [68]*, TGS1* [69], *ORC3* [39], *MAP3K* (N46) [41], *GID1* [70] (Additional file 16). Moreover, an examination of the candidate expression levels revealed some variation between stages and/or reproductive modes. For example, transcript TRpn_185717 which codifies for an ARGONAUTE 104 protein [71], showed a constant higher level of expression in the apomictic genotype during all the developmental stages, while its expression decreased from premeiosis to anthesis in the sexual one (Fig. 7a). Transcript TRpn_57024, with high similarity to a gibberellin receptor (*GID1*), exhibited an insignificant expression in the apomictic genotype at all developmental stages with a peak at anthesis, while it was steadily expressed at low levels in sexual genotypes (Fig. 7b). Two other genes, *ORC3b* (TRpn_33086/TRpn_96407) and *APOSTART2* (TRpn_175767 /TRpn_52614) showed the same expression patterns that exhibited in the in vivo experiments reported in *P. simplex* and *P. pratensis* (Fig. 7c-f) [39, 65].

## Discussion

Apomixis in *Paspalum notatum* is a genetically heritable trait controlled by a single complex-locus (ACL), which shows non-mendelian segregation against apomixis and a strong restriction of recombination involving around 36 M[bp [72–74]. The ACL is localized in a chromosome fragment syntenic to subtelomeric segments of rice chromosomes 2 and 12 and maize chromosomes 1 and 3 [55, 75, 76]. This genomic region displays typical heterochromatin features, like the presence of repetitive elements, gene degeneration and cytosine hypermethylation [75, 77]. The complex genomic topography together with the unavailability of a reference genome have seriously compromised the identification of genes controlling the trait by positional map-based approaches. As a consequence, transcriptomic surveys became essential tools for identifying apomixis-related genes in the species [48, 50].

In the early 2000s, PCR-based methods were used to predict several *P. notatum* transcripts associated with the occurrence of agamospermy [28, 29]. These preliminary amplification analyses, although rather limited, served well to the selection of candidates and the establishment of further functional characterizations that led, finally, to the identification of major components of the apomixis pathway in this species. In particular, the MAP3K-encoding *QGJ* (*QUI-GON JINN*) transcript, initially identified by Laspina et al. (2008) as clone N46, was validated in sexual and aposporous plants through RNA in situ hybridization experiments [41] and functionally classified as an inducer of aposporous embryo sacs (AESs) formation in *Paspalum* RNAi lines [41]. Moreover, *TGS1* (*TRIMETHYLGUANOSINE SYNTHASE 1*) [29] was also confirmed to express differentially in sexual and aposporous ovules by RNA in situ hybridization experiments [69] and classified as an AESs repressor in *Paspalum* antisense lines [78]. The functional association between the apospory induction/repression pathways directed by *QGJ* and *TGS1* is currently under investigation**.**

The development of the first *P. notatum* floral transcriptomes of reference for apomictic and sexual genotypes widened the opportunities for identifying reproductive genes and provided a whole catalogue of full-length molecules related to traits of agronomical interest [31]. However, the scarcity of reliable statistical information due to the absence of replicates was a serious challenge to network interaction predictions and functional analysis preparation [31]. Moreover, since the reference transcriptomes were produced from bulked samples representing different developmental stages, the temporal variation of expression during development remained unexplored at a wide-genome level. Indeed, stage-specific qRT-PCR analyses were carried out for some of the candidates in several genotypes of contrasting reproductive modes, in order to evaluate the chronological evolution of the expression in the course of development. Finally, no information on the antisense/sense orientation of the DETs was available, a fact that partially constrained the elucidation of the mechanisms involved in the sexuality/apomixis transition. In particular, since the differential expression of several retrotransposons [79], long non-coding RNAs (lncRNAs) [40] and pseudogenes [39] was associated with the occurrence of apomixis in *Paspalum*, determining the sense/antisense nature of these groups of transcripts is central to disclose their possible role on molecular mechanisms regulating reproduction.

The set of sequences presented here considerably increases the number of transcripts available from the Roche 454/FLX + reference library [31] and offers information on the expression dynamics for each sequence in the course of development. The GDEA analysis showed 13,205 (68%) and 6147 (32%) transcripts up- and down-regulated during apomixis, providing full-length sequences for most of them and pointing to a greater complexity for the molecular control of agamospermy with respect to sexuality. Meanwhile, the SSDEA study revealed a higher number of DETs than GDEA, a clear indication of expression heterochronicity for a considerable number of transcripts (that is because those genes showing upregulation at different developmental stages in apomictic and sexual plants are classified as ‘non-differential’ in the GDEA). Particularly, a large number of DETs and DEATs was detected at anthesis, which reveals an abundance of exclusive pathways at this stage. Regarding this, chronological differences in the onset of the sexual and apomictic embryonic periods should be taken into account: in apomictic plants, parthenogenesis of the 2n (non-reduced) egg cell starts at the end of megagametogenesis, while in sexual plants, the legitimate n (reduced) egg cell remains quiescent until fertilization [78]. Then, at least a part of the differentially expressed transcripts detected at anthesis might be related alternatively to the induction of parthenogenesis or to embryo development, since, at anthesis, embryos are forming in apomictic plants only. Besides, other genes like those related to pollen-stigma interactions or pollen discharge into the egg cell could also be integrating this particular group of DETs.

We found 11,417 antisense transcripts expressed in flowers, and hundreds of them were differentially represented in sexual and apomictic genotypes. These results confirmed the evidence reported in previous articles, in which both sense and antisense transcripts were detected in apomictic and sexual genotypes at variable representation levels or altered localization [39, 41, 68]. Several of these transcripts, like those homologous to *SERK2*, *MAPK3* and *ORC3* [39, 41, 68] where found here represented by antisense strands and thus, a regulatory mechanism based on complementary hybridization might be associated with them. The confirmation of such a modulation process will require further functional analysis. Interestingly, a methylation-mediated silencing mechanism was reported to control parthenogenesis in other species of the *Paspalum* genus [77]. We detected a higher number of DEATs occurring at anthesis, in comparison with other developmental stages. Moreover, the anthesis DEATs have a tendency (59%) to appear downregulated in apomictic plants. Our results indicate that: 1) antisense-mediated regulatory mechanisms might be particularly active at anthesis in sexual plants; and 2) silencing of a considerable number of DEATs occurs in apomictic plants at this particular stage. It will be interesting to investigate the influence of genome methylation in the down-regulation of DEATs during apomixis, and its consequences on the representation of DETs and the emergence of autonomous embryos in the absence of fertilization.

Two of the main ontology classes previously associated with apomixis in *Paspalum* corresponded to transcription factors [31, 50] and hormones [31, 50, 80]. Here, we identified a number of transcriptional regulators showing differential expression in reproductive organs of apomictic and sexual plants. Some of them, like MYB family members, show heterochronic upregulation in the apomictic and sexual libraries, with expression reaching peaks at different developmental stages. MYB proteins share the conserved MYB DNA-binding domain that is crucial to the control of proliferation and differentiation in several cell types [81]. Besides, upregulation in the apomictic libraries was observed for members of the bHLH, ERF, WRKY, B3, ARF and AP2 family, all of them previously related to plant development through cell division, proliferation and differentiation control [82]. We also identified numerous members of different hormonal pathways, especially the auxin and jasmonate routes, that show differential regulation during apomixis, a fact that had been anticipated when analyzing the representation of miRNAs in reproductive organs of sexual and apomictic plants [80]. Currently several members of these pathways are under functional characterization to determine its role in apomixis development. Besides, the expression characterization for several transcripts previously associated with apomixis provided information on the specific stages these transcripts are modulated and a proof of concept that the use of the catalogue presented here might contribute to a thorough comparison of both reproductive pathways.

The Illumina sequence database reported here represents a detailed chronological characterization of the sense/antisense gene expression landscape in reproductive organs of sexual and apomictic counterparts of the same species. The derived information could be of use in *Paspalum* research programs dealing with gene expression during sexual and asexual seed formation, as well as the molecular breeding of apomixis. Moreover, it will allow the identification of apomixis candidate genes, which could be further characterized in expression and function in other apomictic species. The reported information could not be completely systematized in a single scientific article, since a number of different analyses can be conducted, depending on the need of each specific research project. However, the main use for this tool no doubt will be the comprehensive identification of candidate genes that can be used as molecular markers in apomixis-based breeding programs or to induce asexuality from sexual genetic backgrounds through genetic engineering. In particular, it will allow for rapid discrimination of some of the sequences controlling apomeiosis and parthenogenesis, due to its potential to expose differential expression at specific stages. Such discrimination was impracticable when using the formerly-produced apomictic vs. sexual transcriptomic databases, since their construction invariably involved only one reproductive stage or a bulk of stages. Currently, the *Paspalum* genome is in process of sequencing and assembly in our laboratory and the apospory controlling locus (ACL) is being identified by positioning markers that were fully linked to the trait in former genetic mapping experiments. In this context, mapping the stage-specific candidate sequences exposed here onto the *Paspalum* ACL will help to identify the genomic controllers of apomixis, while RNA in situ hybridization will reveal the precise site of expression in reproductive tissues. Eventually, functional analysis will disclose the reproductive phenotypes that can be induced after up- or down- regulation in precise cell types pointed by the in situ analysis. Moreover, genetic engineering will allow the harnessing of these candidates to reproduce the desired reproductive phenotypes in species of interest, using the appropriate promoters. On another note, an additional application of this database, among many others, will be aimed at clarifying the functionality of the expression originated from the heterochromatic non-recombinant ACL, since mapping the antisense transcripts identified here onto the *Paspalum* genome will reveal which of these transcripts emerge from this particular region.

Difficulties involved in the elucidation of the molecular control of reproduction in *P. notatum* range from the usual complexities of all reproductive systems (the molecular intricacy of the routes involved, the high temporal variation rate, the involvement of cell-specific expression patterns, among others) to those derived from the particular nature of apomictic species, like the evolutionary and physical characteristics of the ACL and the involvement of poorly characterized polyploid heterozygous genomes. Despite all these drawbacks, during the last years, an unprecedented research effort led to detailed characterization of the operational molecular routes for both *P. notatum* reproductive modes, through the establishment of publicly available solid reference transcriptomes [31] and replicate sRNA libraries [80]. Here, we are deepening the characterization of the molecular transcriptional landscape operational in sexual and apomictic plants, by providing a chronological, high yield, orientation-sensitive transcript database covering all reproductive stages from premeiosis to anthesis. We hope that this contribution will serve as a basis to promote future research on the functional mechanisms controlling agamospermy in plants and as a valuable resource for those plant breeders who are focused on the introduction of apomixis technology into their cultivar improvement programs.

## Conclusions

Here we introduce a complete sense/antisense gene expression catalogue from florets of apomictic and sexual *P. notatum*, involving four subsequent reproductive developmental stages, from premeiosis to anthesis. This comprehensive sequence collection quantitatively reveals apomixis vs. sexual heterochronic expression and sense/antisense mediated regulation. In particular, contrasting transcriptional and hormonal control was detected and thoroughly characterized. Our analysis exposed a considerable alteration of sense/antisense expression occurring at the onset of parthenogenesis. The experimental approach used in this work established a set of differentially expressed sequences well beyond the former group of candidates detected in *Paspalum*, which even discriminated the sequence orientation, giving important clues on antisense-mediated transcriptional and post-transcriptional regulation. This dataset will be applied to a more efficient selection of apomixis candidate genes, contributing to the future development of molecular tools for harnessing the trait.

## Methods

### Plant material

The following *P. notatum* genotypes were used for this study: *i*) Q4117, a tetraploid (2n = 4x = 40) natural obligate apomictic accession originated from Southern Brazil [83], and ii) C4-4x, an artificially generated completely sexual induced autotetraploid (2n = 4x = 40) genotype, obtained after chromosome duplication of a sexual diploid plant by colchicine treatment [84]. Vegetative replicates of these plants are being maintained in experimental plots at Instituto de Botánica del Nordeste (IBONE), CONICET-UNNE, (Corrientes, Argentina) and Instituto de Investigaciones en Ciencias Agrarias de Rosario (IICAR), CONICET-UNR (Rosario, Argentina). Q4117 and C4-4x flowering periods overlap (Q4117: November to April; C4-4x: January to April). Flowering conditions are the same for both genotypes. Voucher specimens of this material are kept at the Herbarium CTES-IBONE (publicly available), under deposition numbers: C4-4X (Quarin, C. L. 4260, barcode CTES0541627, cardboard No. 330064); Q4117 (Quarin, C. L. 4117, barcode CTES0541626, cardboard No. 233851).

### RNA isolation

Inflorescences at different stages of the reproductive development were collected from both the Q4117 (apomictic) and C4-4x (sexual) plants. The classification of the reproductive stages was carried out following the protocol described by Laspina et al. [29], by analyzing in parallel both the mega- and microsporogenesis as well as the mega- and microgametogenesis processes. Four stages were considered: (I/II) premeiosis: megaspore mother cells (MMCs) and apospory initials (AIs) are visible in ovules, while tetrads start to appear in anthers; (III) meiosis: uninucleate pollen and female meiosis occurs in the sexual genotype; (IV-VI) postmeiosis: uninucleated/binucleate pollen, and first division of megagametogenesis; and (IV) anthesis: binucleate pollen and mature embryo sacs [29]. Total floral RNA was extracted with the SV RNA Total Isolation Kit (Promega) and quantified using the Quant-iT RiboGreen RNA Reagent and Kit (Invitrogen). Three replicates were established, corresponding to different floral RNA extractions from the same genotypes (Q4117 and C4-4x). The RNA quality was evaluated with RNA 6000 PicoChip (Agilent Bioanalyzer 2100).

### Library preparation and Illumina HiSeq sequencing

Library preparation and sequencing experiments were carried out at Instituto de Agrobiotecnología de Rosario (INDEAR), Rosario, Argentina. Libraries were built using the TruSeq® Stranded mRNA kit (Illumina) starting from 1 μg of total RNA. Procedures for mRNA purification (using oligo-dT hybridization), RNA fragmentation, double-stranded cDNA synthesis, end-adenylation, ligation of adapters and enrichment (amplification) of library fragments were performed following the protocol described in TruSeq® Stranded mRNA Illumina (October 2017). The library quality was checked with the DNA 1000 Kit (Agilent Technologies), using 1 ul of each preparation in a 2100 Bioanalyzer. Libraries resulted in double-stranded DNA fragments with an average size of 260 bp. Three biological samples were processed for each one of the developmental stages (premeiosis, meiosis, postmeiosis, anthesis), for both reproductive modes. Thus, a total of 24 (3 × 4 × 2) libraries were constructed. Before sequencing, the individual libraries were quantified by qPCR (Light Cycler 480 Roche) and normalized to a concentration of 3 nM. One equimolar pool of all libraries was prepared and quantified by qPCR (Light Cycler 480 Roche) using the Qiagen Library Quantification Kit. The pool was used for the generation of clusters in the sequencing cell. A sequencing run was performed by generating paired ends (PE) 2 × 100 bp reads in a HiSeq– Illumina device.

### Bioinformatics methods

Raw reads were de-multiplexed and checked for quality using the FastQC software (http://www.bioinformatics.babraham.ac.uk/projects/fastqc/). Adaptors, duplicated sequences, ambiguous reads, and low-quality reads were removed by using Trim Galore (http://www.bioinformatics.babraham.ac.uk/projects/trim_galore/). The high-quality reads (QC > 30) were used for assembling the transcriptomes with Trinity v2.0.2 [60, 61]. The available Roche 454/FLX + *P. notatum* reproductive transcriptome generated by Ortiz et al. [31] was initially used for a reference-guided assembly with the parameters:“--SS_lib_type RF --normalize_by_read_set --min_contig_length 400”, and --genome_guided_bam. Then, a second de novo assembly (without a reference) was carried out using the default parameters. The four assemblies (apo_over reference, sex_over reference, apo_without reference and sex_without reference) were combined in one file and the non-redundant transcripts were selected using CD-HIT [85]. The quality of the assemblies was measured using QUAST [86]. The raw reads were mapped to the global assembly using Bowtie2 (v 2.3.2.0) [87] and TopHat (v2.1.1) [88]. The transcriptome coverage was evaluated using Benchmarking Universal Single-Copy Orthologs (BUSCO, version 3.0.2) [89, 90] with the following commands: “Python run_BUSCO.py –i sequence_file –o output_name –l lineage –m tran”, “Python generate_plot.py –wd working directory” and the “liliopsida_odb10” dataset. The coding competence for all expressed transcript sequences was predicted using the TransDecoder software (https://github.com/TransDecoder/TransDecoder.wiki.git) with the default parameters (−m 100). For differential expression analysis, RNA-seq reads were analyzed with the Kallisto v.0.44.0 software to determine transcript counts and abundances. The libraries were normalized by size and low-count transcripts were filtered out (< 3 in the three replicas of each library). Differential expression analysis and the corresponding *p*-values were estimated using the Bioconductor software package DESeq2 [91]. The *p*-values attained by the Wald test were corrected for multiple testing using the Benjamini-Hochberg method. The adjusted *p*-values (DESeq2 padj or FDR) thresholds for considering transcripts as DETs/DEATs were < 0.001 and < 0.05 for sense and antisense transcripts, respectively. Moreover, analyses of DETs were restricted to those showing an absolute value of Log_2_FC > |3|. Comparisons of gene expression between modes of reproduction were carried out considering all stages of development (global comparison) or each developmental stage (stage-specific comparison). Venn diagrams were created by using the jvenn online tool/software (http://jvenn.toulouse.inra.fr/app/example.html) [92]. The transcriptome dynamics was analyzed with R: normalized counts were used to execute a hierarchical clustering using a simple euclidean distance metric and a complete linkage method.

### GO analysis and pathway mapping of the *Paspalum notatum* transcriptome

Transcripts were analyzed by their function using BLASTn (https://www.ncbi.nlm.nih.gov) on the NCBI NT database. A similar analysis was performed on the *Oryza sativa* database available at the Gramene webpage (http://www.gramene.org/). The GO numbers were obtained by using ClusterProfiler [93] (http://www.geneontology.org) over the *Arabidopsis* database. Additionally, DETs were submitted to KEGG pathways analysis (KEGG: Kyoto Encyclopedia of Genes and Genomes) (https://www.genome.jp/kegg/ko.html) [94–96] and classified with the single-directional best hit for transcriptional factors (plant transcriptional factors database, http://planttfdb.cbi.pku.edu.cn, http://planttfdb.gao-lab.org) and hormone families (UniVIO: http://univio.psc.riken.jp/, http://hormones.psc.riken.jp/pathway_ja.html). Moreover, critical differentially expressed pathways were analyzed by STRING (https://string-db.org) [62] to infer their possible function and association between them.

## Supplementary Information

**Additional file 1 **Gene coverage of *P. notatum* assemblies estimated with BUSCO. Busco_454: aAnalysis carried out using the Roche-454 reference transcriptome [31]. Busco_TRPN: analysis performed with the Illumina GTA reported here. TRPN stands for Trinity *Paspalum notatum* Assembly (Global Assembly).

**Additional file 2 **GO and KEGG analysis of *P. notatum* GTA. (A-C): 30 most representative GO terms for each category. CC: cellular component. BP: Biological Process. MF: Molecular Function. (D): 30 most represented KEGG pathways. TRPN stands for Trinity *Paspalum notatum* Assembly (Global Assembly).

**Additional file 3.** Sense apomictic vs. sexual differentially expressed transcripts (DETs) derived from the GDEA. IDs, transcripts lengths, Log_2_FCs, p-adjust values and NCBI descriptions (top hit) were included in the table. Positive Log_2_FCs indicate overexpression in apomictic plants. Negative Log_2_FCs indicate overexpression in sexual plants.

**Additional file 4.** Comparative analysis of sense transcript representation in apomictic and sexual libraries. Volcano comparative plots were constructed both globally and separately for each developmental stage. Red dots correspond to DETs at p-adjust < 0.001 and Log_2_FC > ǀ3ǀ. Positive Log_2_FCs indicate overexpression in apomictic plants. Negative Log_2_FCs indicate overexpression in sexual plants.

**Additional file 5.** Heatmap of sense and antisense transcripts showing differential expression between apomictic and sexual libraries at four developmental stages (premeiosis, meiosis, postmeiosis, anthesis). (A): sense transcripts. (B) antisense transcripts. AP1, AP2, AP3: triplicate samples of apomictic premeiotic libraries. AM1, AM2, AM3: triplicate samples of apomictic meiotic libraries. APo1, APo2, APo3: triplicate samples of apomictic postmeiotic libraries. AA1, AA2, AA3: triplicate samples of apomictic anthesis libraries. SP1, SP2, SP3: triplicate samples of sexual premeiotic libraries. SM1, SM2, SM3: triplicate samples of sexual meiotic libraries. SPo1, SPo2, SPo3: triplicate samples of sexual postmeiotic libraries. SA1, SA2, SA3: triplicate samples of sexual anthesis libraries. Repd: reproductive mode.P: premeiosis. M: meiosis. Po: postmeiosis. A: Anthesis.

**Additional file 6.** Antisense apomictic vs. sexual differentially expressed transcripts (DEATs) derived from the GDEA. IDs, transcript lengths, Log_2_FCs, p-adjust values and NCBI descriptions (top hit) were included in the table. Positive Log_2_FCs indicate overexpression in apomictic plants. Negative Log_2_FCs indicate overexpression in sexual plants.

**Additional file 7.** Comparative analysis of antisense transcript representation in apomictic and sexual libraries. Volcano comparative plots were constructed globally and at each developmental stage. Red dots indicate DEATs at p-adjust < 0.05 and Log_2_FC > ǀ3ǀ. Positive Log_2_FCs indicate overexpression in apomictic plants. Negative Log_2_FCs indicate overexpression in sexual plants.

**Additional file 8.** Stage-specific apomictic vs. sexual differential expression analysis for sense transcripts (DETs). Section 1 (S1): premeiosis. Section 2 (S2): meiosis. Section 3 (S3): postmeiosis. Section 4 (S4): anthesis. IDs, transcript lengths, Log_2_FCs, p-adjust values and NCBI descriptions (top hit) and GO annotations were included in the table. Positive Log_2_FCs indicate overexpression in apomictic plants. Negative Log_2_FCs indicate overexpression in sexual plants.

**Additional file 9.** Stage-specific apomictic vs. sexual differential expression analysis for antisense transcripts (DEATs). Section 1 (S1): premeiosis. Section 2 (S2): meiosis. Section 3 (S3): postmeiosis. Section 4 (S4): anthesis. IDs, transcript lengths, Log_2_FCs, p-adjust values and NCBI descriptions (top hit) and GO annotations were included in the table. Positive Log_2_FCs indicate overexpression in apomictic plants. Negative Log_2_FCs indicate overexpression in sexual plants.

**Additional file 10.** KEGG pathways for DETs and DEATs regulated at different stages of sexual and apomictic developments. DETs: several pathways show a differential representation only at a given developmental stage (i. e., the bottom nine molecular routes change only at anthesis). DEATs: Spliceosome and endocytosis DEATs are expressed exclusively at meiosis and anthesis. Several pentose/glucuronate interconversion transcripts are expressed as DEATs only at anthesis.

**Additional file 11 **GO classification and KEGG pathways of common DETs that are differentially expressed across all stages of sexual or apomictic development. (A): cellular components (CC). (B): biological process (BP). (C): molecular function (MF). (D): KEGG pathways: ribosome, carbon metabolism and spliceosome are represented by numerous members at a *p*-value < 0.05.

**Additional file 12.** Transcriptome dynamics. Sections 1 and 2: Matrix of GTA sense (S1) and antisense (S2) transcripts displaying the normalized number of raw reads corresponding to each transcript in each library. Sections 3 and 4: graphics displaying the evolution of expression for particular clusters of sense (S3) and antisense (S4) differentially expressed candidates (DETs and DEATs, respectively) in sexual and apomictic libraries.

**Additional file 13.** List of sequences associated with transcription factors occurring in the GTA. The length of the transcripts, the Log_2_FCs between apo and sex libraries, the p-adjust value, the %ID and the e-value (for the BLASTx top hit) are included for each member of the list.

**Additional file 14 **Classification and expression analysis of transcription factors (TFs) expressed during *P. notatum* floral development. (A): Relative abundance of the identified TF families. (B): Heat map representing the number of upregulated members corresponding to each TF family at four developmental stages in apomictic and sexual genotypes of *P. notatum.* AP: apomixis, premeiosis. AM: apomixis, meiosis. APo: apomixis, postmeiosis. AA; apomixis, anthesis. SP: sexual, premeiosis. SM: sexual, meiosis. SPo: sexual, postmeiosis. SA; sexual, anthesis.

**Additional file 15 **List of transcripts related to plant hormones occurring in the GTA. The length of the transcript, the Log_2_FCs between apo and sex libraries, the p-adjust value, the %ID with *Arabidopsis* transcripts, the similarity e-value and descriptions (for the BLASTx top hit) are included for each member of the list.

**Additional file 16.** List of apomixis candidates and their associated transcripts expression levels, at different developmental stages. Several genes associated with apomixis in previous studies are differentially represented in the current analysis. Identifier: number assigned to the previously characterized apomixis candidate at GenBank/NCBI. DE stages: differential expression detected at each stage, red numbers indicate statistically significant differential expression. The NCBI description of the BLASTx top hits and the GO analysis for each DET was incuded in the last five columns.

## Data Availability

The datasets generated and/or analyzed during the current study are available in the NCBI SRA repository at the following SRA accession: PRJNA511813. This Transcriptome Shotgun Assembly project has been deposited at DDBJ/EMBL/GenBank under the accession GIUR00000000. The version described in this paper is the first version, GIUR01000000. Other dataset(s) supporting the conclusions of this article are included within the article and its additional files. The plant materials used in this study belong to the living germplasm collection of Instituto de Botánica del Nordeste (IBONE), CONICET-UNNE, Corrientes, Argentina. Voucher specimens of this material are kept at the Herbarium CTES-IBONE (publicly available), under deposition numbers: C4-4X (Quarin, C. L. 4260, barcode CTES0541627, cardboard No. 330064); Q4117 (Quarin, C. L. 4117, barcode CTES0541626, cardboard No. 233851).

## Abbreviations

ACL: Apospory controlling locus
AES: Aposporous embryo sac
AI: Apospory initial
AP2: Apetala2
ARF: Auxin response factor
bHLH: Basic helix-loop-helix
BLAST: Basic local alignment search tool
BP: Biological process
bZIP: Basic leucine zipper domain
C2H2: Cys2His2 zinc-finger domain
C3H: Cys3His zinc finger domain
CC: Cellular component
DE: Differentially expressed
DEAT: Differentially expressed antisense transcript
DET: Differentially expressed transcript
ERF: Ethylene response factor
FAR1: Far-red impaired responsive 1
FC: Fold change
FDR: False discovery rate
G2-like: Golden2-like
GDEA: Global differential expression analysis
GO: Gene ontology
GRAS: GAI, RGA, SCR genes family
GTA: Global transcriptome assembly
KEGG: Kyoto encyclopedia of genes and genomes
LBD: Lateral organ boundaries domain
LFY: Leafy
lncRNA: Long non-coding RNA
MAPK: Mitogen-activated protein kinase
MF: Molecular function
miRNA: microRNA
MMC: Megaspore mother cell
mRNA: Messenger RNA
M-type MADS: MCM1, AG, DEFA and SRF genes family
NAC: NAM, ATAF and CUC family
NZZ/SPL: Sporocyteless/nozzle
SAP: Sterile apetala
sRNA: Small RNA
SSDEA: Stage-specific differential expression analysis
TF: Transcription factor
TPM: Transcripts per million, the number of reads per transcript normalized to the library size
TRPN: Trinity *Paspalum notatum* assembly
