# Supplementary figures and images for: A study of the heterochronic sense/antisense RNA representation in florets of sexual and apomictic Paspalum notatum
Source: BMC Genomics. 2021 Mar 16;22:185. doi: 10.1186/s12864-021-07450-3 (PMC7962388; doi:10.1186/s12864-021-07450-3)

## BUSCO Assessment Results

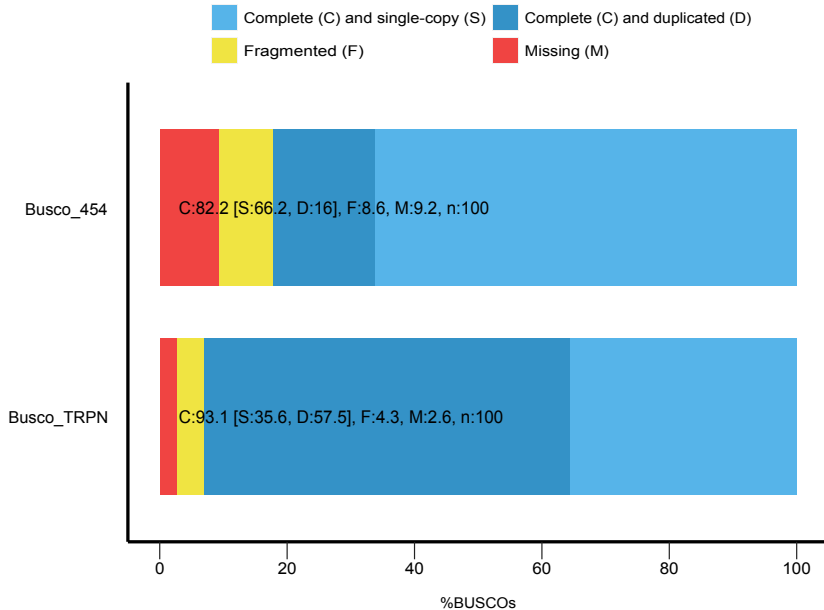

Supplement: Supplementary file 1 — Additional file 1 Gene coverage of P. notatum assemblies estimated with BUSCO. Busco_454: aAnalysis carried out using the Roche-454 reference transcriptome [31]. Busco_TRPN: analysis performed with the Illumina GTA reported here. TRPN stands for Trinity Paspalum notatum Assembly (Global Assembly). [file 12864_2021_7450_MOESM1_ESM.pdf]

(A)

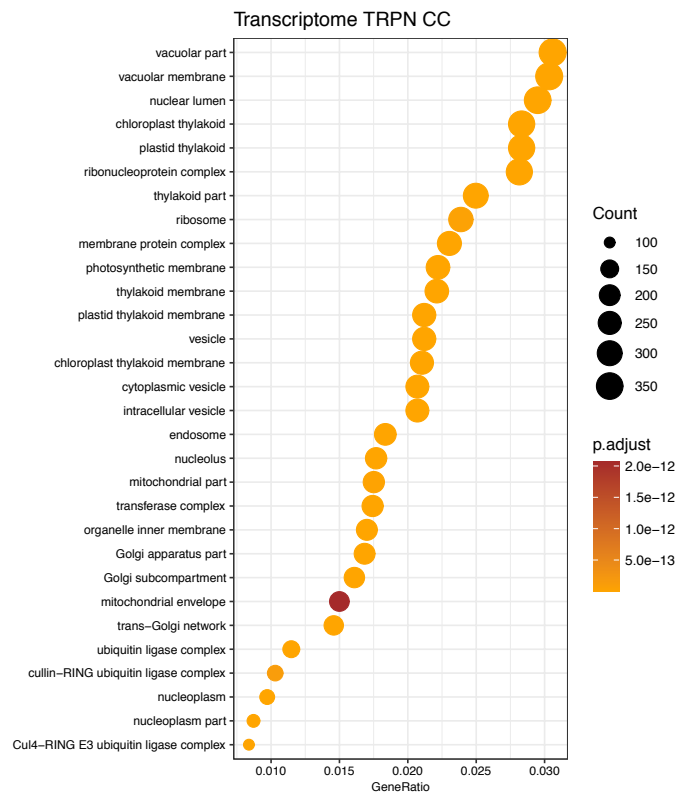

(B)

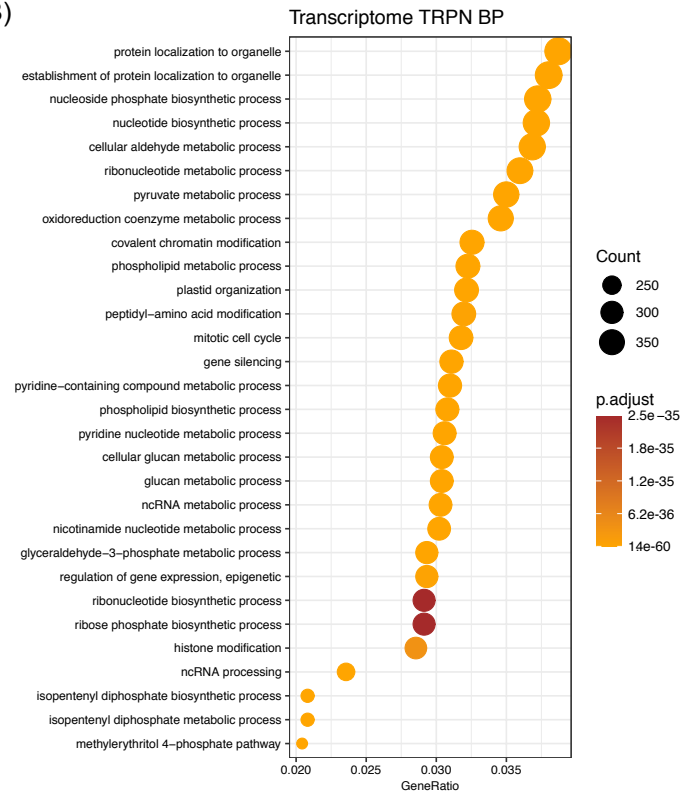

(C)

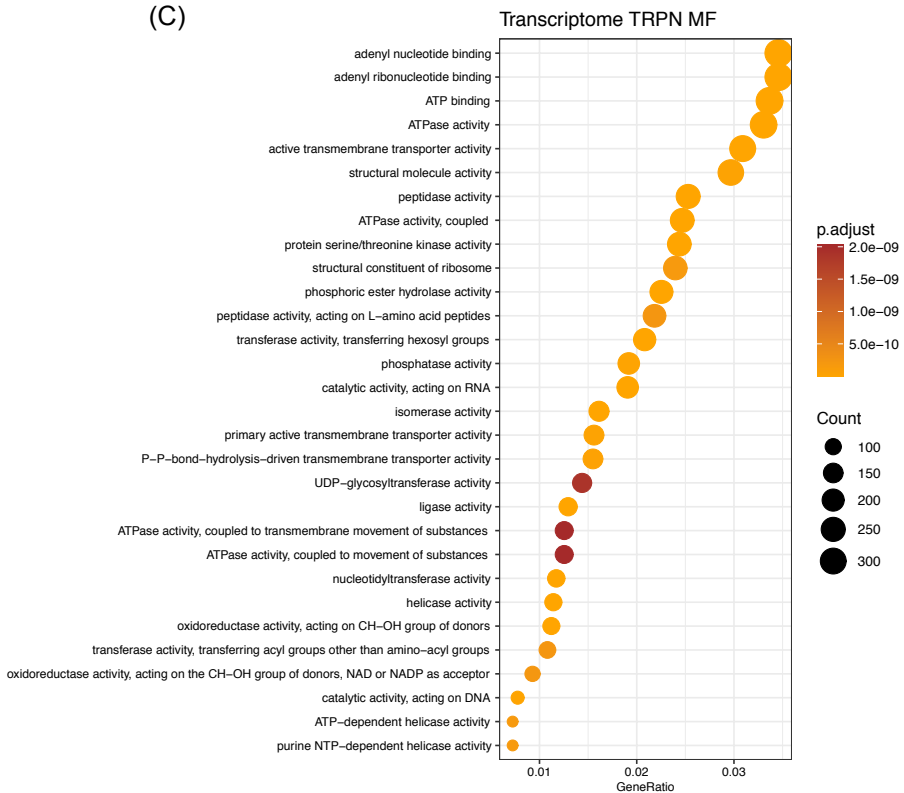

(D)

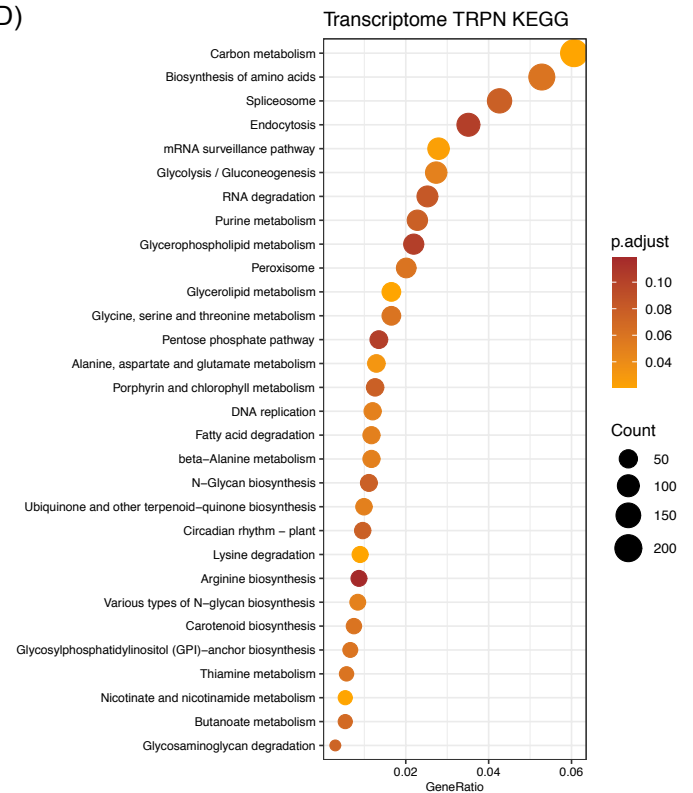

Supplement: Supplementary file 2 — Additional file 2 GO and KEGG analysis of P. notatum GTA. (A-C): 30 most representative GO terms for each category. CC: cellular component. BP: Biological Process. MF: Molecular Function. (D): 30 most represented KEGG pathways. TRPN stands for Trinity Paspalum notatum Assembly (Global Assembly). [file 12864_2021_7450_MOESM2_ESM.pdf]

(A) Apo vs Sex - Global

Log<sub>2</sub> fold change

Total = 188823

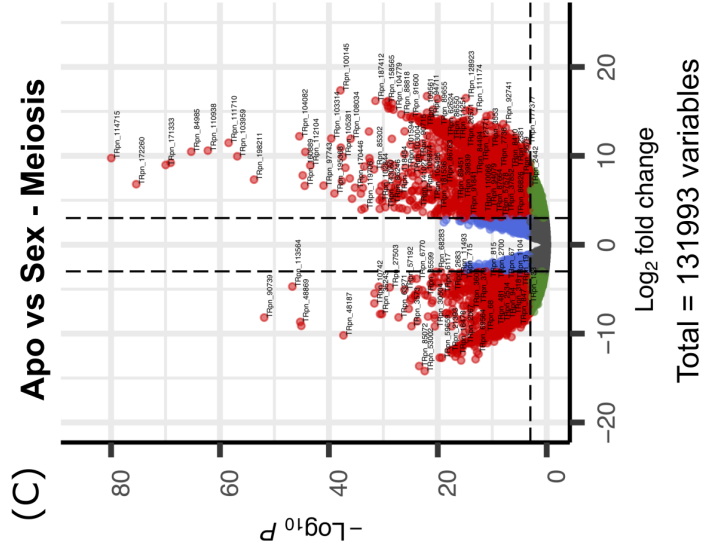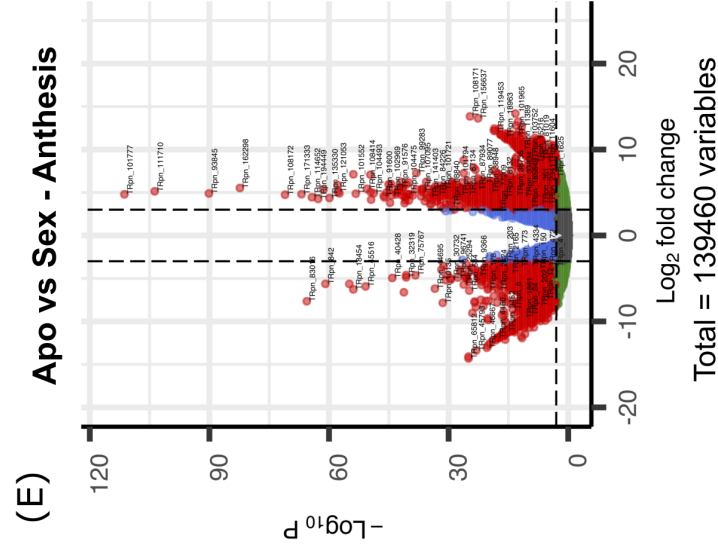

Supplement: Supplementary file 4 — Additional file 4. Comparative analysis of sense transcript representation in apomictic and sexual libraries. Volcano comparative plots were constructed both globally and separately for each developmental stage. Red dots correspond to DETs at p-adjust < 0.001 and Log2FC > ǀ3ǀ. Positive Log2FCs indicate overexpression in apomictic plants. Negative Log2FCs indicate overexpression in sexual plants. [file 12864_2021_7450_MOESM4_ESM.pdf]

(A)

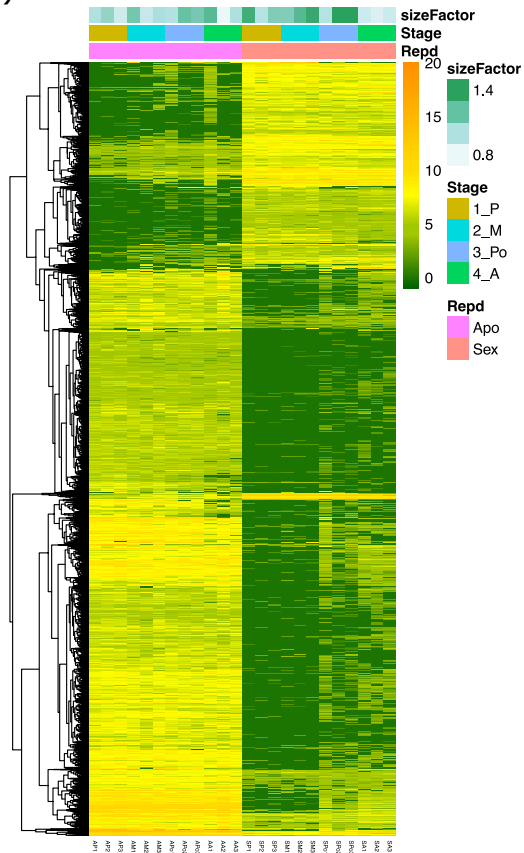

(B)

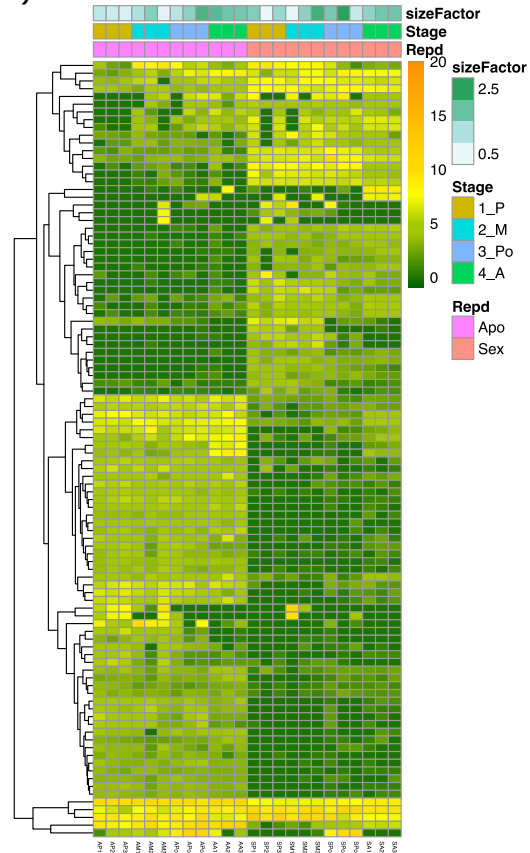

Supplement: Supplementary file 5 — Additional file 5. Heatmap of sense and antisense transcripts showing differential expression between apomictic and sexual libraries at four developmental stages (premeiosis, meiosis, postmeiosis, anthesis). (A): sense transcripts. (B) antisense transcripts. AP1, AP2, AP3: triplicate samples of apomictic premeiotic libraries. AM1, AM2, AM3: triplicate samples of apomictic meiotic libraries. APo1, APo2, APo3: triplicate samples of apomictic postmeiotic libraries. AA1, AA2, AA3: triplicate samples of apomictic anthesis libraries. SP1, SP2, SP3: triplicate samples of sexual premeiotic libraries. SM1, SM2, SM3: triplicate samples of sexual meiotic libraries. SPo1, SPo2, SPo3: triplicate samples of sexual postmeiotic libraries. SA1, SA2, SA3: triplicate samples of sexual anthesis libraries. Repd: reproductive mode.P: premeiosis. M: meiosis. Po: postmeiosis. A: Anthesis. [file 12864_2021_7450_MOESM5_ESM.pdf]

# KEGG Pathways by Stages - DET

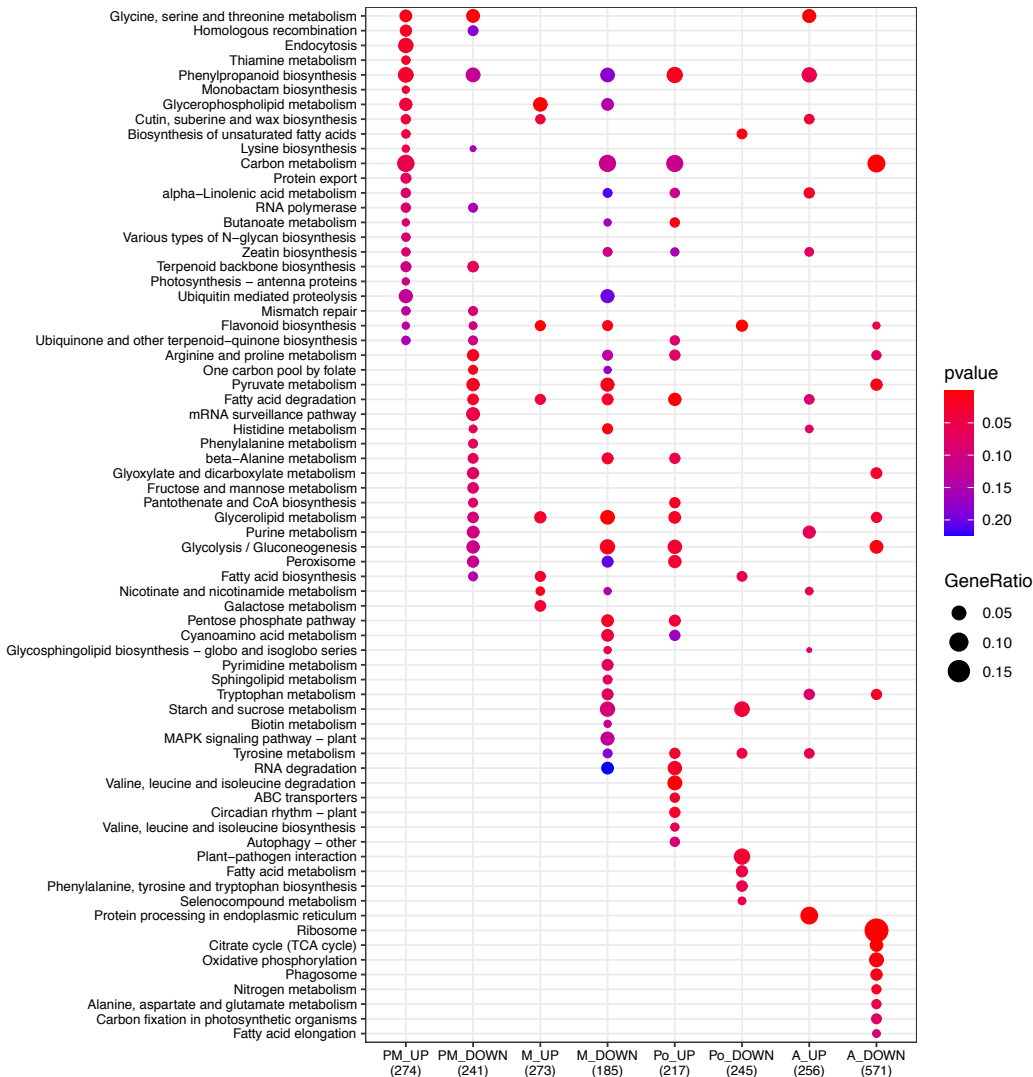

# KEGG pathways by stages - DEAT

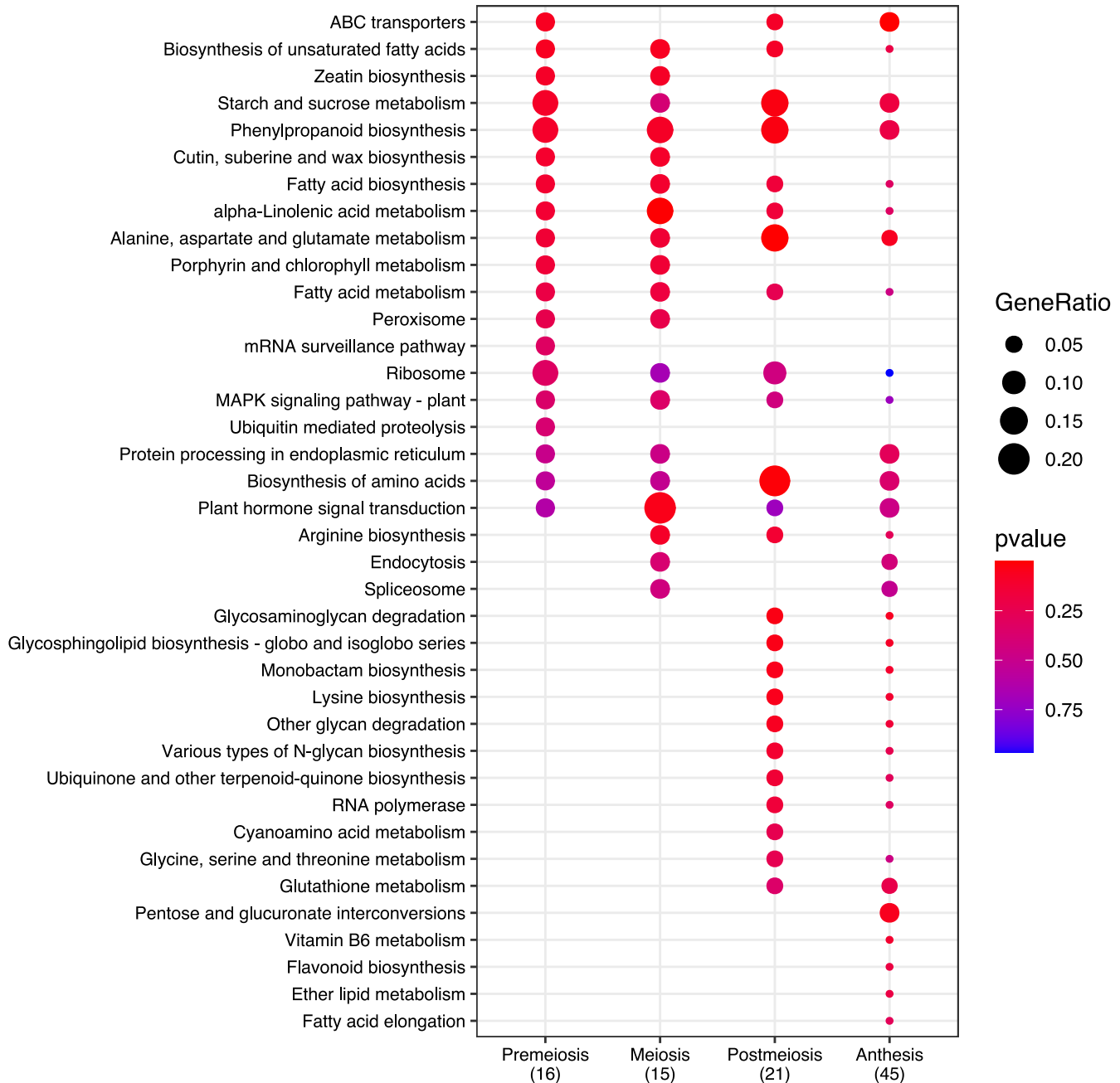

Supplement: Supplementary file 10 — Additional file 10. KEGG pathways for DETs and DEATs regulated at different stages of sexual and apomictic developments. DETs: several pathways show a differential representation only at a given developmental stage (i. e., the bottom nine molecular routes change only at anthesis). DEATs: Spliceosome and endocytosis DEATs are expressed exclusively at meiosis and anthesis. Several pentose/glucuronate interconversion transcripts are expressed as DEATs only at anthesis. [file 12864_2021_7450_MOESM10_ESM.pdf]

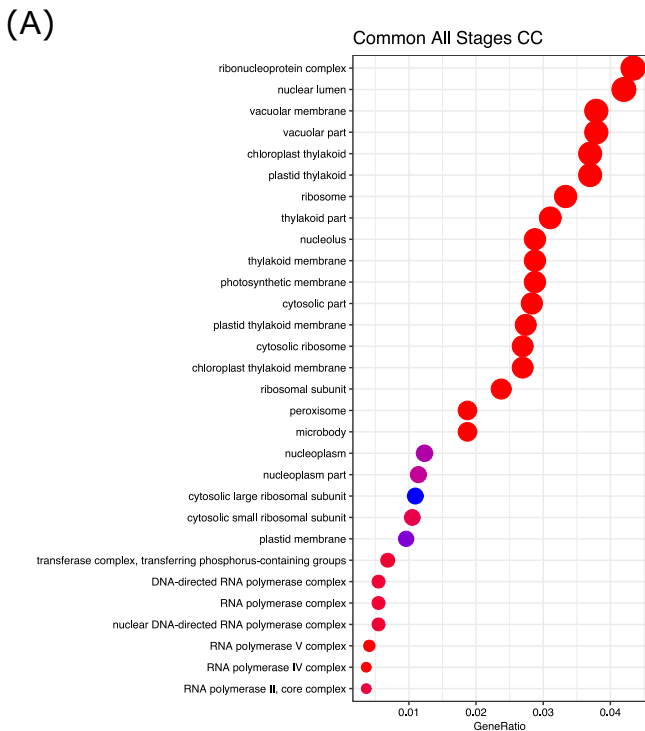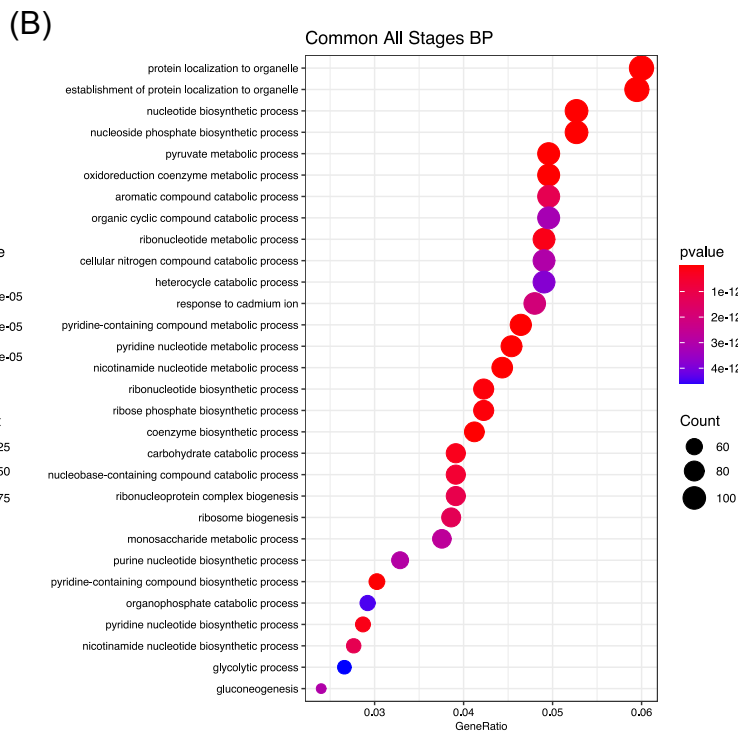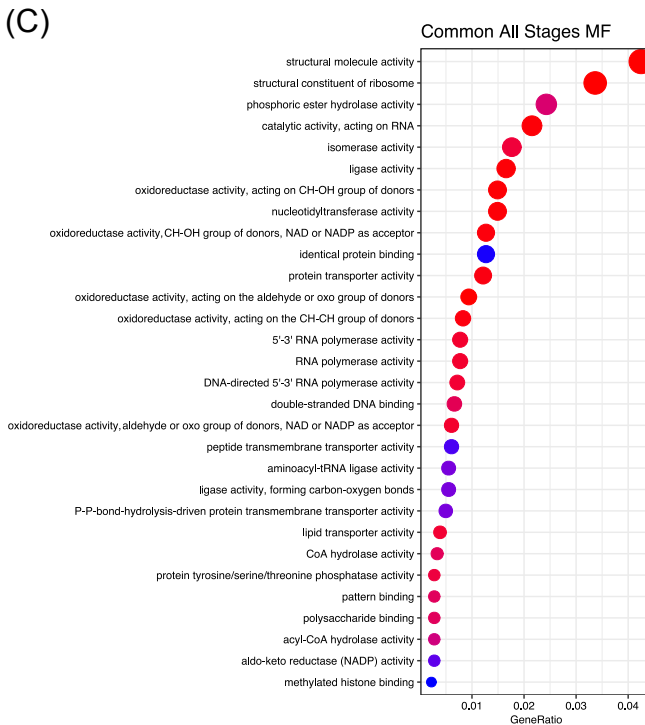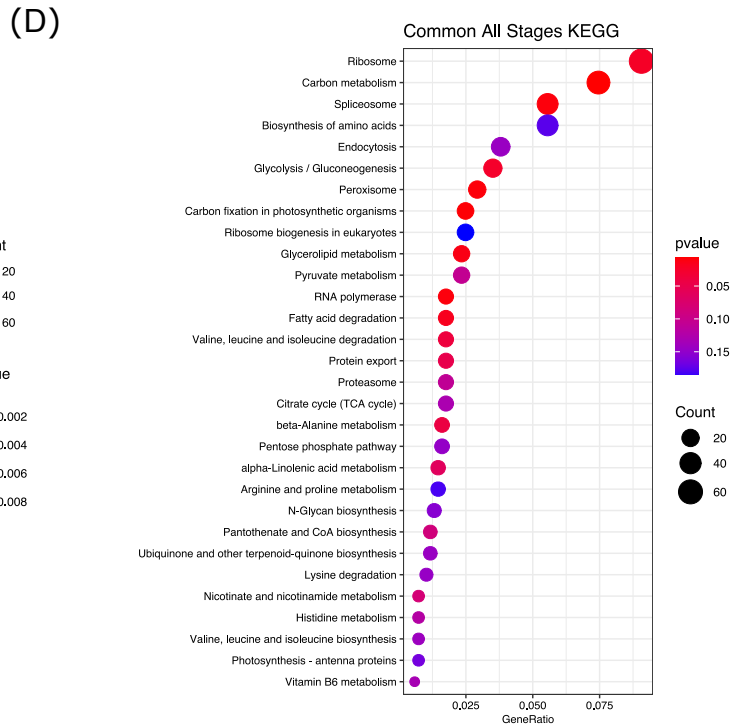

Supplement: Supplementary file 11 — Additional file 11 GO classification and KEGG pathways of common DETs that are differentially expressed across all stages of sexual or apomictic development. (A): cellular components (CC). (B): biological process (BP). (C): molecular function (MF). (D): KEGG pathways: ribosome, carbon metabolism and spliceosome are represented by numerous members at a p-value < 0.05. [file 12864_2021_7450_MOESM11_ESM.pdf]

(A)

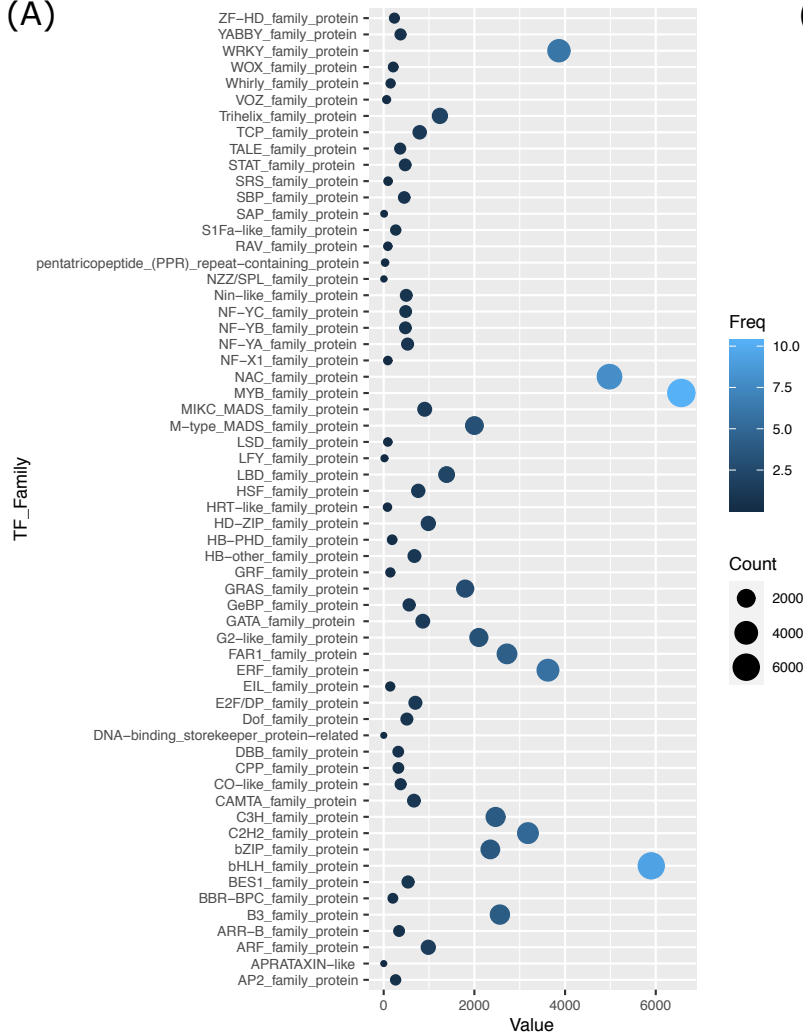

(B)

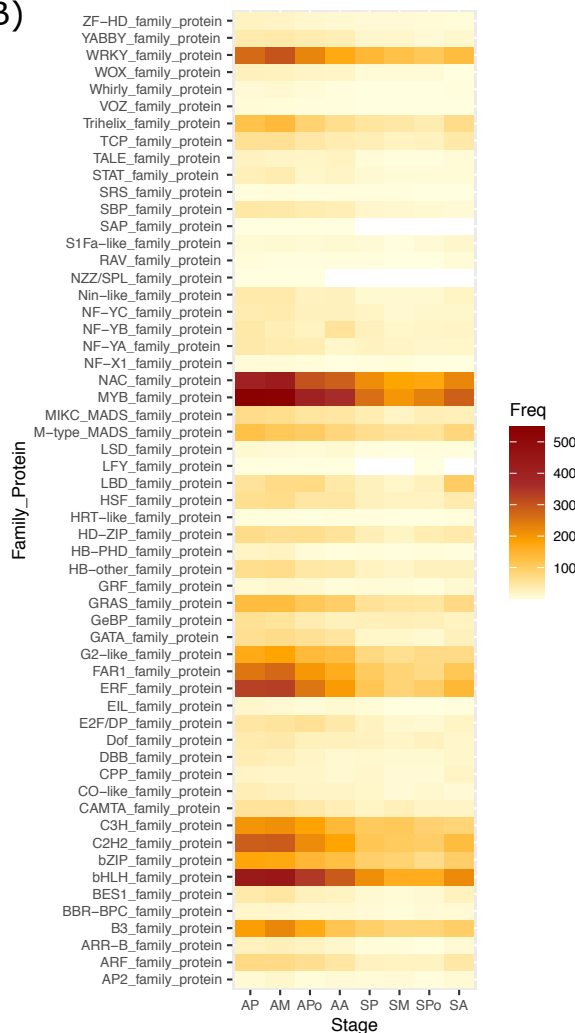

Supplement: Supplementary file 14 — Additional file 14 Classification and expression analysis of transcription factors (TFs) expressed during P. notatum floral development. (A): Relative abundance of the identified TF families. (B): Heat map representing the number of upregulated members corresponding to each TF family at four developmental stages in apomictic and sexual genotypes of P. notatum. AP: apomixis, premeiosis. AM: apomixis, meiosis. APo: apomixis, postmeiosis. AA; apomixis, anthesis. SP: sexual, premeiosis. SM: sexual, meiosis. SPo: sexual, postmeiosis. SA; sexual, anthesis. [file 12864_2021_7450_MOESM14_ESM.pdf]
