# Supplementary material for: A study of the heterochronic sense/antisense RNA representation in florets of sexual and apomictic Paspalum notatum
Source: BMC Genomics. 2021 Mar 16;22:185. doi: 10.1186/s12864-021-07450-3 (PMC7962388; doi:10.1186/s12864-021-07450-3)

Antisense Transcripts

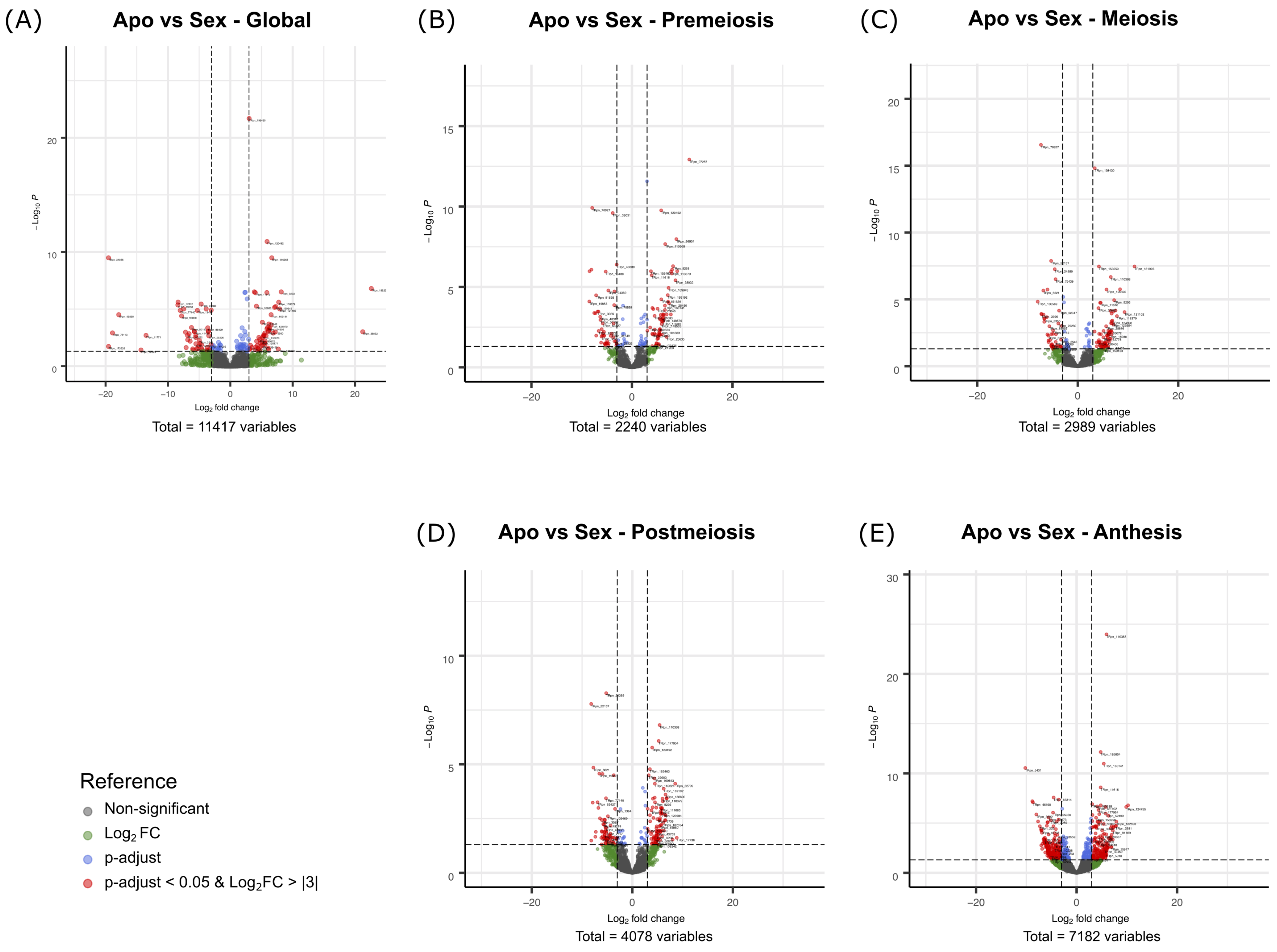

Supplement: Supplementary file 7 — Additional file 7. Comparative analysis of antisense transcript representation in apomictic and sexual libraries. Volcano comparative plots were constructed globally and at each developmental stage. Red dots indicate DEATs at p-adjust < 0.05 and Log2FC > ǀ3ǀ. Positive Log2FCs indicate overexpression in apomictic plants. Negative Log2FCs indicate overexpression in sexual plants. [file 12864_2021_7450_MOESM7_ESM.pdf]
